# Intelligent Circular Polymer Additive Manufacturing: From Recycling Pathways to Lifecycle Engineering

**DOI:** 10.3390/polym18172161

**Published:** 2026-09-04

**Authors:** Francisco J. G. Silva, Filipa Pacheco, Naiara P. V. Sebbe, André Pedroso, Arnaldo G. Pinto

**Affiliations:** 1 CIDEM, ISEP, Polytechnic of Porto, Rua Dr. António Bernardino de Almeida, 4249-015 Porto, Portugal; 1211904@isep.ipp.pt (F.P.); napvs@isep.ipp.pt (N.P.V.S.); afvpe@isep.ipp.pt (A.P.); agp@isep.ipp.pt (A.G.P.); 2 LAETA, Associate Laboratory for Energy, Transports and Aerospace (LAETA-INEGI), Rua Dr. Roberto Frias 400, 4200-465 Porto, Portugal; 3 FEUP—Faculty of Engineering, University of Porto, Rua Dr. Roberto Frias, 4200-465 Porto, Portugal

**Keywords:** intelligent circular manufacturing, lifecycle engineering, design for circularity, polymer additive manufacturing, circular economy, digital material passports, polymer recycling, sustainable manufacturing

## Abstract

Polymer Additive Manufacturing (AM) offers substantial opportunities for material-efficient and distributed production, yet its transition towards genuine circularity remains constrained by cumulative material degradation, fragmented recovery strategies, and the limited integration of lifecycle, environmental, economic, and industrial considerations. This critical systematic review examines circularity in polymer AM beyond conventional end-of-life recycling by integrating material behaviour, manufacturing-induced evolution, degradation mechanisms, lifecycle performance and value retention, recovery pathways, and sustainability assessment within a unified systems perspective. Following a PRISMA-based selection process, 3214 records were progressively screened to a final corpus of 175 peer-reviewed studies published between 2015 and 2025. The evidence demonstrates that polymer circularity is not an intrinsic material property, but an emergent lifecycle outcome governed by polymer chemistry, manufacturing history, cumulative degradation, functional-value retention, waste-stream quality, recovery technology, infrastructure, and environmental and economic conditions. Based on this synthesis, the review introduces Intelligent Circular Polymer Additive Manufacturing (ICPAM), a lifecycle-wide framework integrating Design for Circularity, degradation-aware manufacturing, adaptive recovery, digital intelligence, industrial implementation, and continuous feedback. ICPAM further incorporates multi-criteria decision support for selecting context-dependent circular strategies and a qualitative/semi-quantitative maturity assessment for identifying lifecycle bottlenecks and implementation priorities. The resulting framework shifts polymer AM circularity from reactive waste management towards proactive lifecycle engineering, while recognizing that emerging regenerative materials and digital technologies still require substantial industrial validation before their full circular potential can be realized.

## 1. Introduction

Additive Manufacturing (AM) has evolved from a rapid prototyping technology into a mature manufacturing platform capable of producing functional, high-value components for demanding sectors including aerospace, biomedical engineering, automotive, and energy [1]. The combination of unprecedented geometric freedom, material efficiency, decentralized production, and digital manufacturing has positioned polymer AM as one of the enabling technologies of Industry 4.0 and sustainable manufacturing [2,3,4]. Consequently, AM is frequently portrayed as an environmentally preferable alternative to conventional subtractive and formative manufacturing processes, primarily because it minimizes material waste during fabrication and enables lightweight, functionally optimized components [3,4].

However, this sustainability narrative remains fundamentally incomplete. Most environmental assessments continue to focus predominantly on manufacturing-stage benefits, while the end-of-life (EoL) behaviour of AM-produced polymers receives comparatively limited attention [5]. As polymer AM expands from prototyping towards large-scale industrial production, increasing volumes of discarded components, failed prints, support structures, contaminated powders and post-processing residues are being generated, transforming waste management into one of the principal challenges limiting the long-term sustainability of the technology [6,7].

Unlike conventional polymer processing, where waste streams are generally homogeneous, centralized and relatively easy to recycle, polymer AM generates highly fragmented, chemically heterogeneous and process-dependent waste streams [6]. Material composition is frequently complicated by pigments, reinforcing fibres, functional fillers, soluble support materials, photoinitiators and multi-material architectures [8]. Moreover, each manufacturing process subjects the polymer to a unique thermal and mechanical history, producing irreversible molecular modifications that strongly influence its subsequent recyclability. Consequently, AM waste cannot be considered simply as discarded thermoplastic material; rather, it constitutes a new class of engineered waste whose recycling behaviour is intrinsically governed by the interaction between polymer chemistry, manufacturing history and component architecture [9].

From a materials science perspective, repeated thermal exposure during printing and reprocessing progressively accelerates hydrolysis, thermo-oxidative degradation, chain scission, crosslinking and molecular rearrangement, resulting in continuous deterioration of molecular weight, rheological behaviour and mechanical performance [10,11]. These degradation mechanisms are particularly pronounced in widely used thermoplastics such as PLA, ABS and PA12, whereas photopolymer resins employed in Vat Photopolymerization technologies remain fundamentally incompatible with conventional recycling owing to their permanently crosslinked molecular networks [12,13]. Consequently, although numerous polymer AM materials are frequently classified as technically recyclable, their practical reintegration into high-value manufacturing cycles remains severely constrained.

This apparent contradiction highlights a broader scientific misconception. Recyclability is often interpreted as an intrinsic material property, whereas industrial circularity is, in reality, an emergent systems property resulting from the combined influence of material chemistry, manufacturing-induced degradation, waste logistics, recycling technology, economic viability and regulatory infrastructure [14]. Therefore, evaluating polymer sustainability exclusively through material recyclability provides an incomplete representation of the true environmental performance of additive manufacturing.

Although previous reviews have examined sustainable manufacturing, polymer recycling, circular economy strategies or individual AM technologies, these topics have largely been addressed independently. The literature still lacks a comprehensive framework capable of integrating degradation mechanisms, manufacturing processes, recycling pathways, environmental assessment and industrial implementation into a coherent understanding of circular polymer additive manufacturing [15]. This fragmentation hinders the identification of the governing mechanisms that ultimately determine whether theoretically recyclable polymers can effectively participate in closed-loop manufacturing systems [16].

Despite the growing body of literature addressing sustainable additive manufacturing, polymer recycling, and circular economy strategies, current knowledge remains highly fragmented. Existing reviews predominantly examine individual manufacturing processes, specific polymer families, or isolated recycling technologies, with comparatively limited attention devoted to the complex interactions between manufacturing-induced degradation, recycling efficiency, material performance, and industrial implementation. As a result, the mechanisms governing the transition from theoretical recyclability to effective circularity remain insufficiently understood.

A fundamental limitation of the current literature is the widespread assumption that recyclability is an intrinsic property of polymeric materials. In practice, however, the circular performance of AM components emerges from the interaction of multiple interconnected factors, including polymer chemistry, processing history, degradation mechanisms, waste heterogeneity, recycling technology, logistics, environmental impacts, and economic feasibility. These interactions have rarely been analysed within a unified scientific framework.

To address this gap, the present review adopts a systems-level perspective that integrates materials science, manufacturing engineering, recycling technologies, life-cycle assessment, and circular economy principles. Rather than providing a descriptive overview of existing recycling approaches, the review critically examines the governing mechanisms limiting circular polymer additive manufacturing while identifying future technological and industrial directions.

Accordingly, the principal scientific contributions of this review are:

- Establishing an integrated conceptual framework linking manufacturing processes, degradation mechanisms, recycling pathways, and circularity performance.
- Critically comparing the recyclability of the principal polymer families employed in additive manufacturing using a unified analytical perspective.
- Identifying the dominant degradation mechanisms controlling material performance throughout successive recycling cycles.
- Evaluating the gap between theoretical recyclability and effective industrial circularity from technical, environmental, and economic viewpoints.
- Defining future research priorities for enabling scalable closed-loop polymer additive manufacturing through Design for Circularity, advanced recyclable polymers, digital traceability, and dedicated recycling infrastructures.

The critical analysis performed throughout this review indicates that achieving true circularity in polymer additive manufacturing requires a paradigm that extends beyond conventional recycling-oriented approaches. Based on the synthesis of the reviewed evidence, this work introduces the concept of Intelligent Circular Polymer Additive Manufacturing (ICPAM), which describes an integrated manufacturing ecosystem where polymer design, additive manufacturing, degradation control, adaptive recycling, digital intelligence, lifecycle management, and industrial implementation operate as interconnected components of a unified engineering system.

This concept provides the organizing framework that guides the analysis developed throughout the remainder of this review. Throughout this review, the term Intelligent Circular Polymer Additive Manufacturing (ICPAM) is used to describe the integrated systems-level paradigm proposed in this work, serving as the conceptual framework that connects the analytical dimensions, scientific evidence, and engineering implications discussed in the following sections. Within the ICPAM perspective, circularity is no longer interpreted as an end-of-life recovery strategy but as a continuously managed lifecycle property that is designed, monitored, optimized, and regenerated throughout successive manufacturing cycles.

For the benefit of the reader, the remainder of this paper is organized as follows. Section 2 describes the review methodology, the literature-selection procedure, analytical framework, data extraction, and evidence-quality assessment. Section 3 synthesizes the current state of circular polymer additive manufacturing, covering polymer behaviour, manufacturing-induced effects, degradation mechanisms, recycling and recovery pathways, and environmental and economic performance. Section 4 develops the ICPAM framework and its lifecycle-wide decision-support architecture, including Design for Circularity, degradation-aware manufacturing, intelligent recycling, multi-criteria decision-making, qualitative and semi-quantitative maturity assessment, and industrial implementation. Section 5 discusses the broader scientific and industrial implications of the proposed framework, while Section 6 summarizes the principal conclusions, limitations, and future research directions.

## 2. Methods

### 2.1. Review Design and Scientific Framework

This study was designed as a critical systematic review integrating the methodological rigor of the Preferred Reporting Items for Systematic Reviews and Meta-Analyses (PRISMA) framework with a systems-oriented analytical approach specifically developed for polymer additive manufacturing (AM). Unlike conventional systematic reviews, which primarily summarize published evidence, the present work aims to establish a comprehensive scientific interpretation of the mechanisms governing polymer recyclability throughout the entire additive manufacturing lifecycle.

The review was conceived under the premise that recyclability should not be interpreted as an intrinsic material characteristic, but rather as an emergent systems property resulting from the interaction between polymer chemistry, manufacturing history, degradation mechanisms, recycling technologies, environmental performance, and industrial implementation. Consequently, the analytical framework adopted in this study extends beyond traditional literature synthesis by examining the causal relationships connecting these interdependent dimensions. Thus, the scientific framework was structured around six interconnected dimensions that collectively describe the lifecycle behaviour and circular performance of polymer AM systems:

- Material Behaviour, addressing the intrinsic physicochemical and molecular attributes that determine material stability, processability, degradation susceptibility, and recovery potential.
- Manufacturing-Induced Effects, examining how additive manufacturing conditions modify material structure, thermal history, defects, degradation state, and subsequent lifecycle performance.
- Lifecycle Performance and Value Retention, addressing durability, reliability, service life, repairability, maintainability, reuse potential, property retention, and material traceability as mechanisms for preserving functionality before recycling becomes necessary.
- Recycling and Recovery Pathways, evaluating mechanical, chemical, solvent-based, regenerative, and other recovery strategies according to material condition, technical feasibility, property retention, and potential for reintegration into subsequent manufacturing cycles.
- Circularity and Sustainability Performance, integrating material conservation, environmental performance, lifecycle costs, recovery efficiency, and material value retention to determine whether circular strategies generate effective system-level benefits.
- Industrial and Digital Integration, addressing scalability, standardization, qualification, regulatory compliance, reverse logistics, digital traceability, artificial intelligence, and industrial infrastructure required to implement circular polymer additive manufacturing at scale.

Rather than operating independently, these dimensions form an interconnected lifecycle architecture in which the output of each dimension conditions the performance of subsequent stages while system-level sustainability and industrial data provide feedback for continuous optimization. This interdependence constitutes the operational foundation of the proposed ICPAM framework.

Accordingly, the review develops an integrated conceptual framework linking manufacturing processes, material degradation, recycling strategies, and circularity performance. This framework provides the scientific basis for the critical interpretation presented throughout the subsequent sections and establishes a coherent structure for evaluating the current state of knowledge while identifying future directions for the development of sustainable polymer additive manufacturing systems.

**Figure 1:**
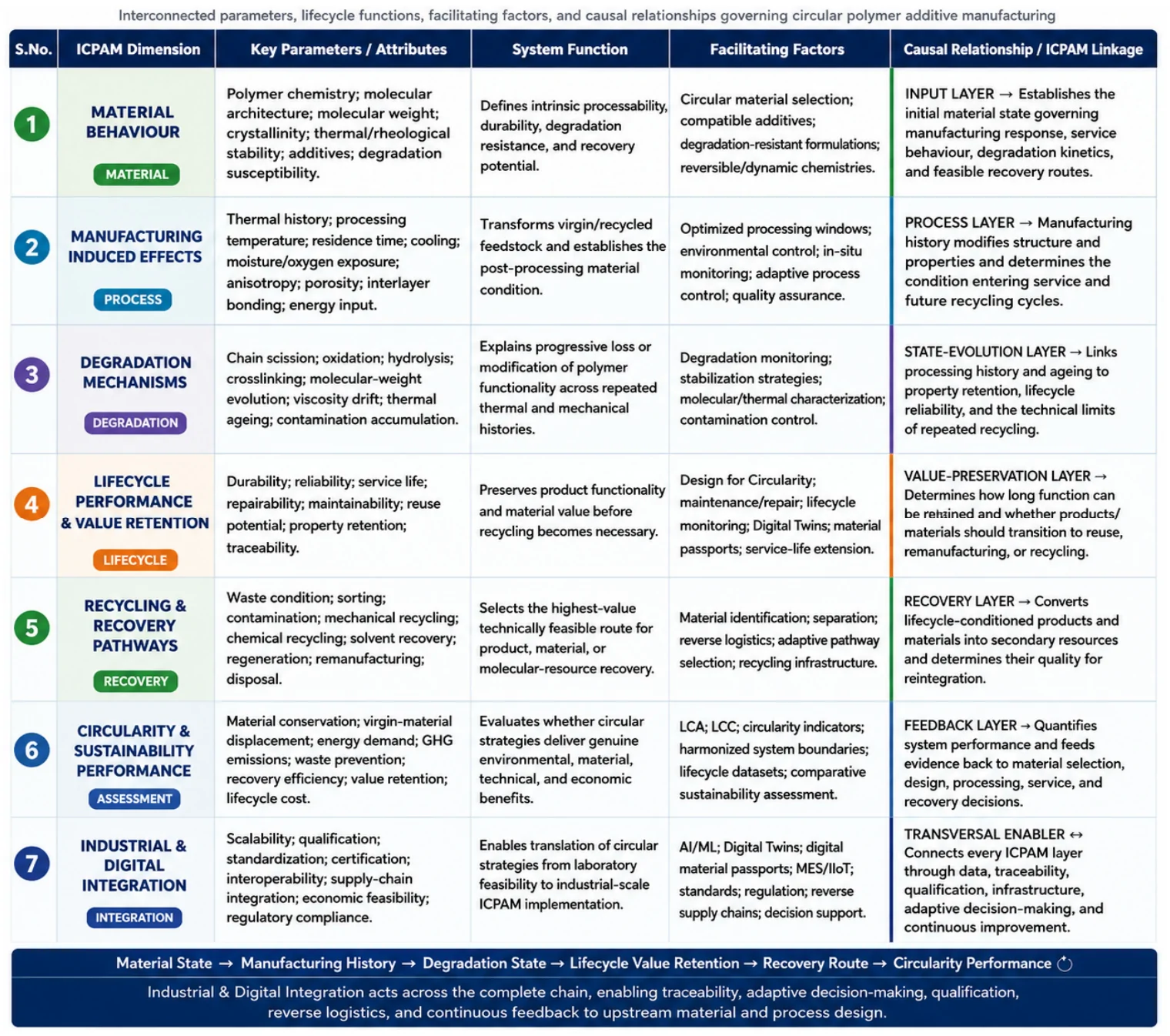
Functional architecture and interrelationships of the ICPAM framework.

To operationalize the proposed Intelligent Circular Polymer Additive Manufacturing (ICPAM) framework, Figure 1 translates these six dimensions into a functional systems architecture by identifying their principal parameters and attributes, system functions, facilitating factors, and interrelationships. Rather than treating the dimensions as independent analytical categories, the revised structure explicitly shows how material behaviour, manufacturing-induced evolution, lifecycle value retention, recovery strategies, sustainability performance, and industrial/digital integration interact to determine the circular performance of polymer additive manufacturing systems.

The relationships represented in Figure 1 should not be interpreted as a strictly linear sequence, but as an interconnected lifecycle architecture. Material behaviour establishes the initial conditions governing manufacturing-induced material evolution, while successive processing and service histories determine degradation mechanisms and the resulting capacity to retain functionality and material value. This condition subsequently governs the feasibility and effectiveness of reuse, remanufacturing, recycling, or other recovery pathways. Circularity and sustainability performance then provide a feedback mechanism for upstream material-design, product-design, and manufacturing decisions. Industrial and Digital Integration acts transversally across the entire architecture, enabling traceability, lifecycle data exchange, adaptive decision-making, qualification, reverse logistics, and continuous system optimization. As summarized in Figure 1, the proposed analytical architecture extends beyond conventional systematic review methodologies by explicitly linking each scientific dimension to the overarching ICPAM framework. This integrated organization establishes a coherent methodological basis for the literature search, critical analysis, and systems-level synthesis presented in the following chapters, ensuring that the review remains conceptually consistent from evidence acquisition to the formulation of future engineering perspectives.

**Figure 2:**
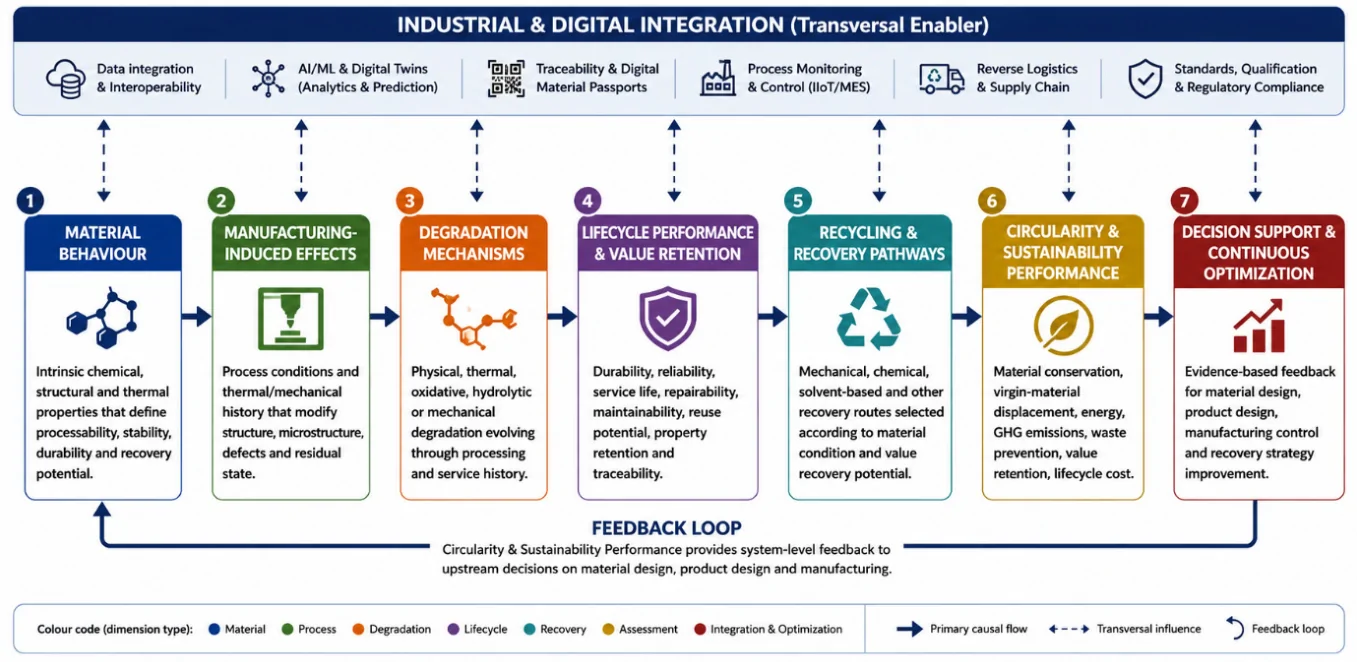
Conceptual representation of the functional relationships, transversal integration, and feedback mechanisms within the ICPAM framework.

To complement the functional architecture summarized in Figure 1 and Figure 2 illustrates the interrelationships among the ICPAM dimensions and their integration across the polymer lifecycle. Rather than representing circularity as a linear sequence ending in recycling, the framework connects material behaviour, manufacturing-induced effects, degradation mechanisms, lifecycle performance and value retention, recovery pathways, and circularity and sustainability performance within a closed-loop architecture. Industrial and digital integration operates transversally across these dimensions, enabling traceability, monitoring, data exchange, and adaptive decision-making throughout the lifecycle.

As illustrated in Figure 2, ICPAM is inherently recursive rather than linear. Material and manufacturing decisions determine subsequent degradation and value-retention behaviour, which in turn condition the technical feasibility and effectiveness of reuse, remanufacturing, recycling, and other recovery pathways. Circularity and sustainability performance close the loop by feeding lifecycle evidence back into upstream material, product, and process decisions, while industrial and digital integration provides the transversal infrastructure required for traceability, qualification, adaptive decision support, and continuous system optimization. This closed-loop logic shifts polymer AM circularity from an end-of-life intervention towards a lifecycle-engineering strategy centred on preserving functionality and material value for as long as technically, environmentally, and economically feasible.

### 2.2. Literature Search Strategy

A comprehensive and reproducible literature search strategy was developed to systematically identify the scientific evidence addressing polymer recyclability within the context of Additive Manufacturing (AM). The search protocol was designed to maximize database coverage while ensuring methodological transparency and minimizing selection bias.

To guide the systematic evidence synthesis and ensure alignment between the literature-selection process and the objectives of the review, five research questions (RQs) were formulated:

RQ1. How do polymer chemistry, feedstock characteristics, and additive manufacturing conditions influence material degradation and property retention across successive processing cycles?

RQ2. Which manufacturing-induced degradation mechanisms most critically affect the recyclability, reprocessability, and long-term functional performance of polymers used in additive manufacturing?

RQ3. How effective are the available reuse, remanufacturing, mechanical recycling, chemical recycling, and emerging recovery pathways in preserving material functionality and value across successive lifecycle loops?

RQ4. Which technical, environmental, economic, and circularity indicators are required to evaluate whether polymer AM systems achieve genuine lifecycle benefits beyond reductions in energy consumption or waste generation alone?

RQ5. How can material behaviour, manufacturing history, degradation, lifecycle performance, recovery pathways, sustainability assessment, and digital/industrial enablers be integrated into a lifecycle-wide decision-support framework for circular polymer additive manufacturing?

These research questions established the analytical scope of the review and guided the definition of the search strings, eligibility criteria, screening procedure, data-extraction categories, and subsequent evidence synthesis. RQ1 and RQ2 primarily address the material–process–degradation relationships governing repeated polymer processing; RQ3 examines the capacity of different circular pathways to retain functionality and material value; RQ4 extends the assessment towards environmental, economic, and system-level circularity performance; and RQ5 integrates the resulting evidence into the proposed ICPAM architecture. Accordingly, the literature-selection methodology was designed not merely to identify publications addressing polymer recycling, but to capture evidence across the complete material–manufacturing–degradation–performance–recovery–circularity chain.

**Figure 3:**
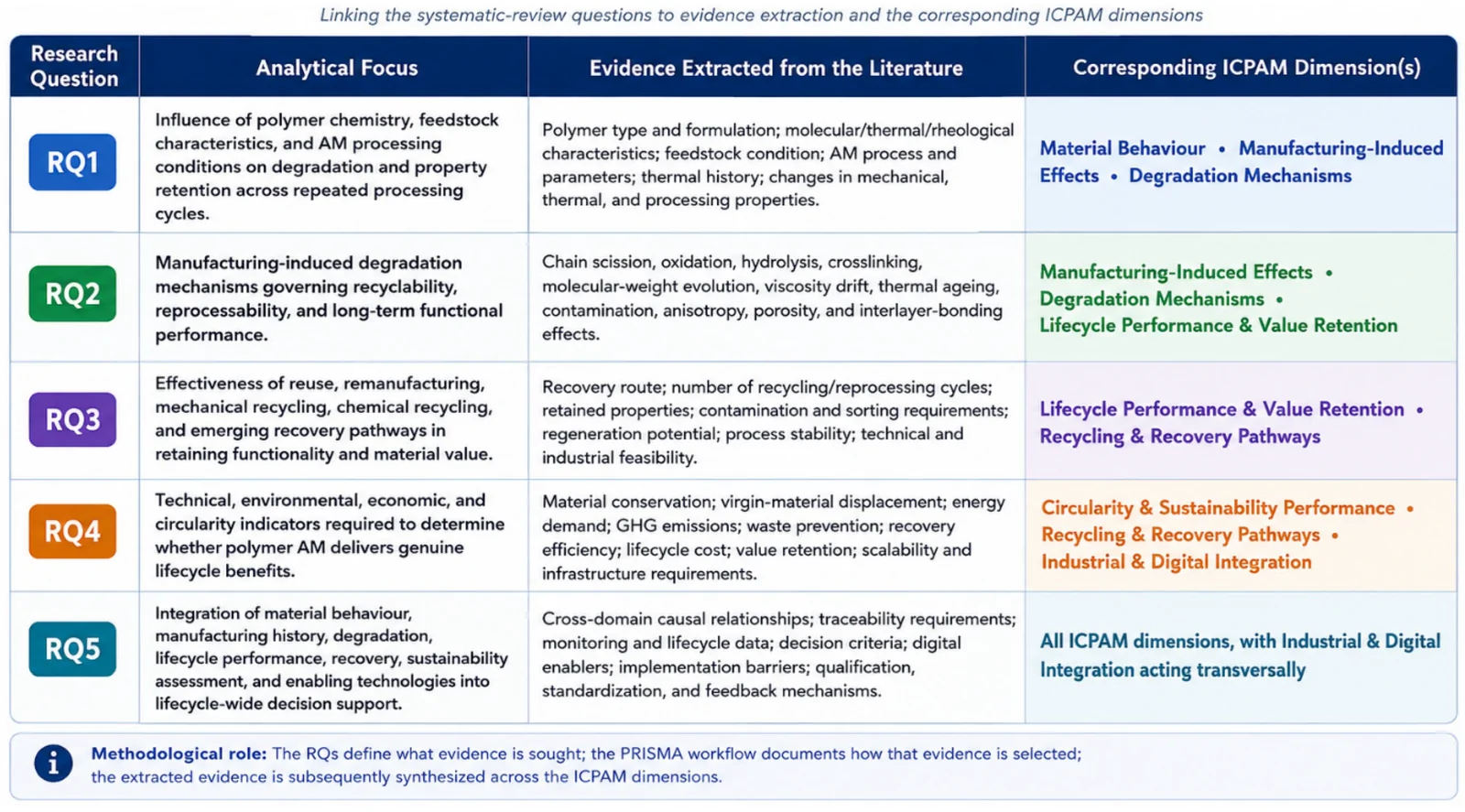
Mapping of the research questions to the analytical focus, evidence-extraction categories, and corresponding ICPAM dimensions.

To make this methodological correspondence explicit, Figure 3 maps each research question onto its principal analytical focus, the categories of evidence extracted from the selected literature, and the ICPAM dimensions to which that evidence contributes. This mapping establishes traceability between the questions guiding the review, the information systematically extracted from the literature, and the subsequent construction of the integrated framework.

As shown in Figure 3, the research questions were intentionally formulated to progress from material- and process-level phenomena towards increasingly integrated lifecycle considerations. RQ1 and RQ2 establish the material–manufacturing–degradation basis of the analysis, RQ3 extends this evidence towards functionality retention and recovery pathways, and RQ4 incorporates environmental, economic, material-conservation, and industrial performance. RQ5 subsequently integrates these evidence streams at system level, providing the methodological basis for the lifecycle-wide and closed-loop architecture embodied in ICPAM.

The literature survey was conducted across four internationally recognized scientific databases: Scopus, Web of Science Core Collection, ScienceDirect (Elsevier), and MDPI. These databases were selected because they collectively provide broad coverage of materials science, polymer engineering, additive manufacturing, circular economy, and sustainability research, while ensuring access to high-quality peer-reviewed publications.

The search strategy was structured around three complementary conceptual domains representing the fundamental dimensions of the present review:

- Additive Manufacturing Technologies: “additive manufacturing”, “3D printing”, “Fused Deposition Modeling”, “FDM”, “Fused Filament Fabrication”, “FFF”, “Selective Laser Sintering”, “SLS”, “Stereolithography”, “SLA”, “Digital Light Processing”, “DLP”, “Material Jetting”.
- Polymeric Materials: “polymer”, “plastic”, “thermoplastic”, “thermoset”, “photopolymer”, “resin”, “filament”, and “powder”.
- Circularity and Recycling: “recycling”, “mechanical recycling”, “chemical recycling”, “closed-loop recycling”, “circular economy”, “life cycle assessment”, “LCA”, “reprocessing”, “material recovery”, “remanufacturing”, and “Design for Recycling”.

Boolean operators (AND/OR) were employed to combine these conceptual domains, enabling the identification of publications simultaneously addressing manufacturing technologies, polymer materials, and end-of-life management. Additional manual screening of reference lists from highly cited review papers and seminal publications was performed to identify relevant studies not captured through database indexing, thereby improving the completeness of the review.

The search covered publications released between January 2015 and December 2025, a period corresponding to the industrial maturation of polymer additive manufacturing and the growing integration of circular economy principles into manufacturing research. Although earlier studies established the technological foundations of polymer AM, the selected timeframe captures the transition from rapid prototyping toward industrial production, where recyclability and sustainability became central scientific and industrial concerns.

To ensure the scientific quality of the evidence base, only peer-reviewed journal articles and full conference papers presenting original experimental results, quantitative analyses, or comprehensive review contributions were considered. This strategy prioritised scientifically validated evidence while maintaining broad coverage of the multidisciplinary research landscape surrounding circular polymer additive manufacturing.

**Figure 4:**
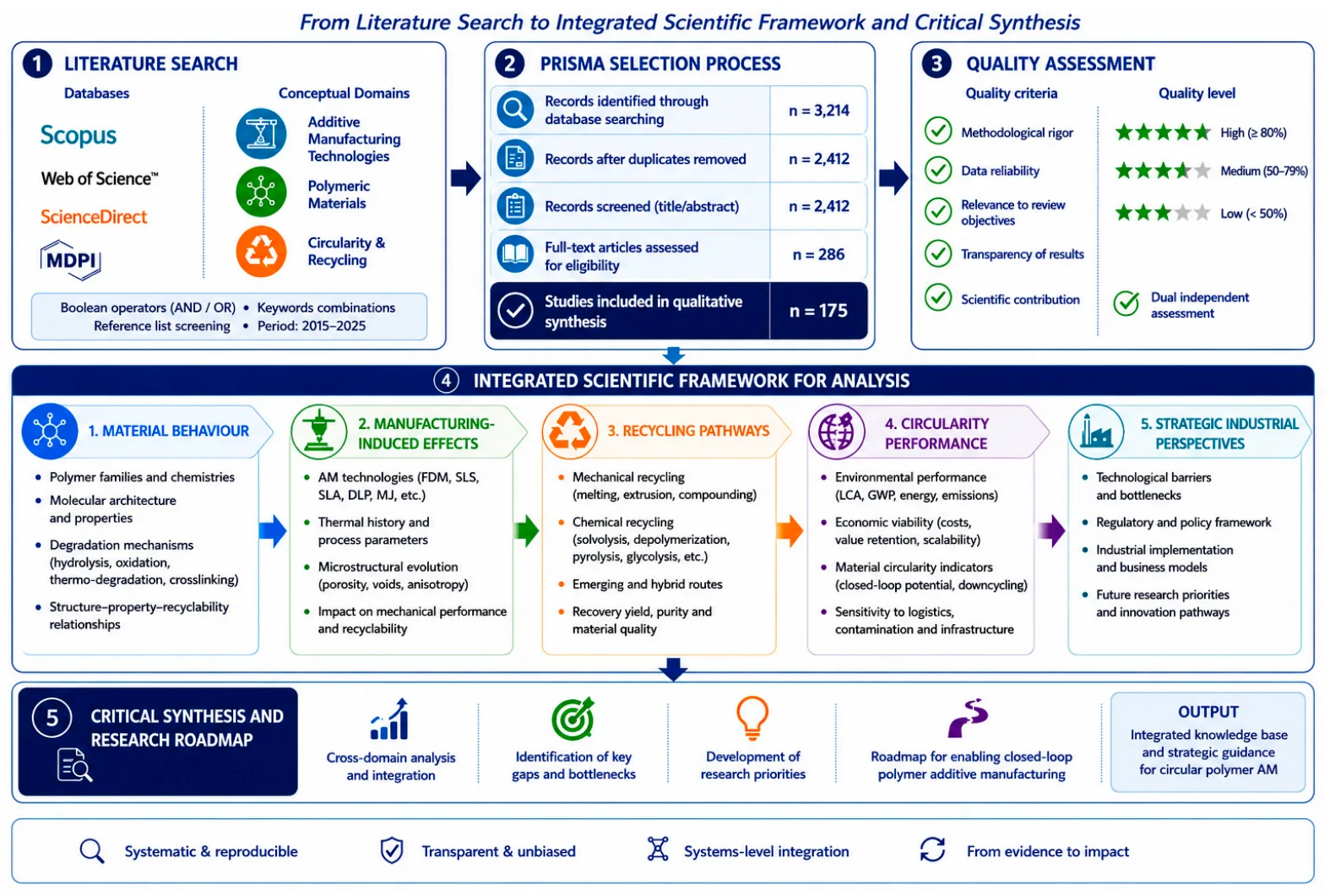
Adopted methodological architecture for the systematic and critical analysis of polymer recyclability in additive manufacturing.

Figure 4 illustrates the methodological architecture adopted in this critical systematic review. Beyond the conventional PRISMA workflow, the proposed framework integrates literature identification, quality assessment, and a systems-level analytical structure that connects material behaviour, manufacturing-induced effects, recycling pathways, circularity performance, and strategic industrial perspectives. This integrated methodology ensures that the subsequent analysis is conducted within a coherent scientific framework, enabling a critical interpretation of polymer recyclability throughout the entire additive manufacturing lifecycle.

This methodological architecture establishes the scientific foundation of the review by ensuring a transparent, reproducible, and integrated evaluation of the available evidence. The resulting framework guides the critical analysis presented throughout the following sections, enabling a systems-level interpretation of circular polymer additive manufacturing.

### 2.3. Eligibility Criteria

To ensure the scientific robustness and relevance of the review, explicit eligibility criteria were established before the literature screening process. The selection protocol was designed to include studies providing direct experimental evidence, quantitative analyses, or comprehensive scientific discussions on the recyclability of polymer materials employed in Additive Manufacturing (AM), while excluding publications whose scope did not contribute to the objectives of the present review.

Studies were considered eligible when they fulfilled all of the following criteria:

- Publication in peer-reviewed scientific journals or full peer-reviewed conference proceedings;
- Publication between January 2015 and December 2025;
- Primary focus on polymer-based additive manufacturing technologies, including Fused Deposition Modeling (FDM), Fused Filament Fabrication (FFF), Selective Laser Sintering (SLS), Stereolithography (SLA), Digital Light Processing (DLP), Material Jetting (MJ), Binder Jetting (BJ), or closely related polymer AM processes;
- Investigation of recyclability, degradation mechanisms, recycling technologies, material recovery, circular economy strategies, Life Cycle Assessment (LCA), or end-of-life management of AM-produced polymer components;
- Presentation of experimental results, quantitative performance evaluation, validated modelling approaches, or critical review evidence directly relevant to polymer circularity.

Conversely, publications were excluded whenever they:

- Focused exclusively on metallic, ceramic, or composite additive manufacturing without addressing polymer recyclability;
- Investigated virgin material processing without considering recycling, degradation, or end-of-life management;
- Presented purely conceptual sustainability discussions unsupported by technical evidence;
- Consisted of editorials, patents, dissertations, technical reports, book chapters, abstracts, or non-peer-reviewed publications;
- Duplicated results already reported in more comprehensive journal articles.

Special attention was given to studies addressing polymer degradation mechanisms, molecular structure evolution, material characterization, recycling performance, and environmental assessment, as these topics constitute the scientific foundation required for understanding circularity in polymer additive manufacturing. Whenever multiple publications reported substantially overlapping experimental datasets, the most comprehensive and technically detailed study was retained in order to avoid duplication of evidence.

The eligibility criteria were established to maximize the scientific quality, reproducibility, and comparability of the reviewed literature while ensuring that the final corpus accurately represented the current state of knowledge regarding polymer recyclability across the complete additive manufacturing lifecycle.

### 2.4. Study Selection

Following the application of the predefined eligibility criteria, the retrieved publications underwent a structured multi-stage selection procedure to ensure methodological consistency and scientific relevance. The selection process followed the Preferred Reporting Items for Systematic Reviews and Meta-Analyses (PRISMA) guidelines while incorporating an additional critical appraisal aimed at identifying studies capable of providing meaningful evidence for the systems-level analysis developed in this review.

The screening procedure consisted of four sequential stages, as depicted in Figure 2. Subsequently, titles and abstracts were independently screened to eliminate publications that were clearly unrelated to polymer additive manufacturing, recycling technologies, circular economy strategies, or material degradation mechanisms. The remaining publications were then subjected to full-text assessment, during which their methodological consistency, technical relevance, and direct contribution to the objectives of the review were critically evaluated, allowing the elimination of the ones out of the main focus of this stud. Finally, only studies satisfying all predefined eligibility criteria were retained for the qualitative synthesis, resulting in 175 publications being used.

Special attention was devoted to avoiding the inclusion of redundant evidence. When multiple publications reported substantially overlapping datasets or successive developments of the same experimental work, the most comprehensive and technically detailed study was selected, thereby minimizing duplication while preserving the breadth of scientific evidence.

Rather than relying exclusively on publication metrics or citation counts, study selection prioritized the scientific relevance and methodological robustness of the reported evidence. Consequently, publications were retained according to their ability to contribute to the understanding of the interactions between material characteristics, additive manufacturing processes, degradation phenomena, recycling pathways, and circularity performance.

**Figure 5:**
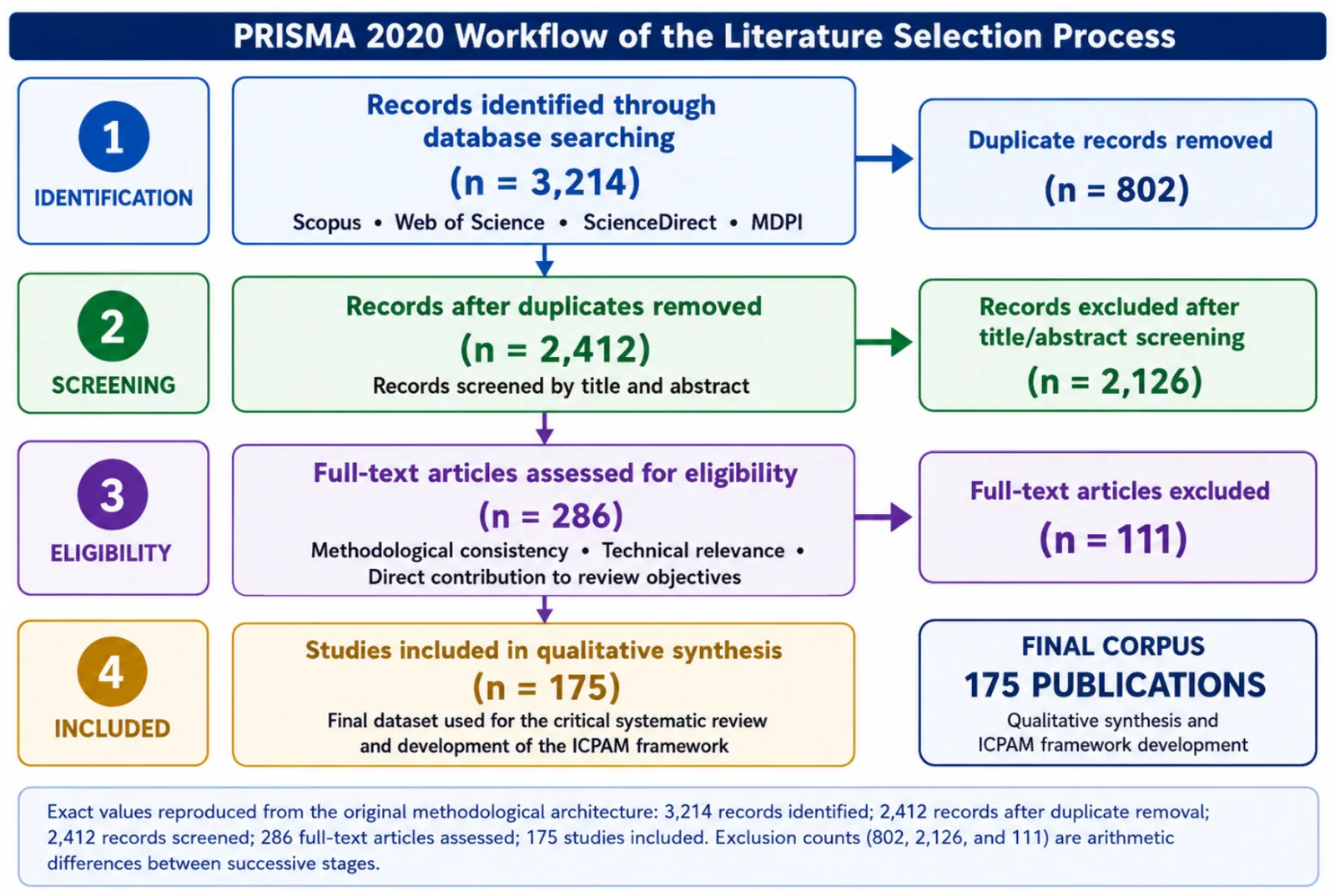
PRISMA 2020 workflow for identification, screening, eligibility assessment, and selection of the 175 publications included in the systematic evidence synthesis.

The identification and screening procedure was subsequently structured according to the PRISMA 2020 workflow (Table S1). As summarized in Figure 5, the database search initially identified 3214 records. After removal of 802 duplicate records, 2412 publications proceeded to title and abstract screening. Of these, 286 publications were retained for full-text eligibility assessment, based on their alignment with the predefined scope and research questions. Following full-text evaluation, 111 publications were excluded, resulting in a final corpus of 175 studies included in the qualitative synthesis and used to support the development of the ICPAM framework.

The progressive reduction from 3214 initially identified records to 175 included studies reflects the deliberately focused scope of the review. Eligibility was determined not by the isolated occurrence of terms related to additive manufacturing or recycling, but by the capacity of each study to provide relevant evidence for at least one of the research questions and to contribute to understanding the relationships among material behaviour, manufacturing-induced degradation, lifecycle performance, recovery, and circularity. The resulting corpus therefore constitutes the evidence base from which the cross-domain relationships and methodological gaps underlying the ICPAM framework were identified.

### 2.5. Data Extraction and Quality Assessment

Following the study selection process, a standardized data extraction protocol was applied to ensure the systematic collection and organization of information from all eligible publications. The protocol was specifically designed to capture the multidisciplinary nature of circular polymer additive manufacturing, allowing direct comparison between studies employing different polymer families, additive manufacturing technologies, recycling strategies, and sustainability assessment approaches.

For each study, information was extracted according to six complementary analytical categories:

- Manufacturing characteristics, including additive manufacturing technology, feedstock configuration, processing parameters, and fabrication conditions;
- Material characteristics, including polymer family, chemical composition, molecular architecture, fillers or reinforcements, and relevant physicochemical properties;
- Degradation mechanisms, comprising thermal, thermo-oxidative, hydrolytic, mechanical, and chemical degradation, together with their effects on molecular structure, crystallinity, viscosity, and mechanical performance;
- Recycling strategy, including mechanical recycling, chemical recycling, feedstock regeneration, solvent-based recovery, and emerging recycling technologies, as well as the corresponding processing conditions and reported material recovery efficiencies;
- Performance indicators, including mechanical, thermal, rheological, morphological, and physicochemical properties before and after recycling, together with recyclability limits and property retention over successive processing cycles;
- Sustainability and lifecycle indicators, including Life Cycle Assessment (LCA) parameters, energy demand, greenhouse gas emissions, resource efficiency, economic feasibility, scalability, and other environmental or industrial performance metrics whenever available.

Within the sustainability dimension, the analysis considered the LCA-related parameters most consistently reported across the selected studies, including cumulative energy demand, greenhouse-gas emissions or global warming potential, virgin material consumption, waste generation and diversion, transportation requirements, and material recovery or recycling efficiency. Where available, water consumption and other environmental impact categories reported by the original studies were also considered. However, the lifecycle sustainability assessment adopted in this review was intentionally broader than environmental LCA alone. Material conservation was examined through indicators such as virgin-material displacement, material recovery, waste prevention, reuse/recycling potential, and material value retention, while economic aspects, when reported, were considered from a Life Cycle Costing (LCC) or broader techno-economic perspective rather than treated as environmental LCA indicators. End-of-life alternatives, including reuse, recycling, material recovery, downcycling, and final disposal, were also critically examined according to their technical feasibility and capacity to retain material value. From the broader Life Cycle Engineering (LCE) perspective, the analysis additionally considered property retention after reprocessing, feasible reuse/recycling cycles, process scalability, reverse-logistics requirements, and recycling-infrastructure availability. Because the reviewed studies employed heterogeneous system boundaries, functional units, datasets, and assessment methodologies, these parameters were used for critical comparative interpretation rather than direct numerical aggregation or meta-analysis.

To ensure that the synthesized evidence was scientifically robust, each publication was subsequently subjected to a qualitative quality assessment based on four complementary dimensions:

- Methodological rigor, evaluating the clarity of the experimental design, reproducibility of the procedures, and adequacy of material characterization techniques.
- Technical relevance, assessing the direct contribution of each study to the understanding of polymer degradation, recycling behaviour, and circular additive manufacturing.
- Evidence strength, considering the consistency of experimental results, validation methods, quantitative analyses, and reliability of the reported conclusions.
- Scientific contribution, evaluating the originality of the work, its relevance to the advancement of circular polymer additive manufacturing, and its potential to support the integrated analytical framework developed in this review.

Rather than adopting a numerical scoring methodology, studies were critically interpreted according to their scientific quality, methodological transparency, and relevance to the objectives of the review. This qualitative approach was considered more appropriate given the substantial heterogeneity of the available literature, which encompasses multiple additive manufacturing processes, polymer systems, degradation phenomena, recycling technologies, and sustainability assessment methodologies.

The extracted information was subsequently synthesized using the integrated analytical framework proposed in Section 2.6, enabling the identification of consistent scientific trends, causal relationships, technological bottlenecks, and emerging research opportunities across the complete polymer additive manufacturing lifecycle.

### 2.6. Analytical Framework

The analytical framework developed in this review was designed to move beyond conventional literature synthesis by establishing a systems-oriented interpretation of polymer recyclability in Additive Manufacturing (AM). Rather than analysing individual studies independently, the selected evidence was integrated into a unified conceptual structure capable of identifying the scientific mechanisms governing circular material performance throughout the complete additive manufacturing lifecycle.

The framework was established under the premise that circularity cannot be adequately explained through isolated analyses of material properties or recycling technologies. Instead, the recyclability of AM polymers emerges from the dynamic interaction of multiple interdependent factors operating across different spatial and temporal scales. Consequently, the analytical methodology adopted in this review considers polymer circularity as a systems-level phenomenon governed by coupled material, manufacturing, environmental, and industrial processes.

To capture these interactions, the reviewed literature was interpreted according to four sequential analytical layers representing the progressive evolution of polymer materials from manufacturing to end-of-life recovery:

Layer I—Material Fundamentals

The first analytical layer examines the intrinsic characteristics of polymeric materials, including molecular architecture, chemical composition, crystallinity, thermal behaviour, rheological properties, and physicochemical stability. These intrinsic properties establish the initial conditions governing degradation susceptibility and recycling potential.

Layer II—Manufacturing-Induced Material Evolution

The second layer evaluates the modifications introduced during additive manufacturing. Particular attention is devoted to thermal history, repeated melting cycles, cooling rates, residual stresses, anisotropy, porosity, interlayer bonding, and process-induced microstructural evolution. These manufacturing effects determine the initial degradation state of the material before entering any recycling pathway.

Layer III—Recycling-Induced Degradation

The third layer analyses the physical and chemical transformations occurring during successive recycling operations. Mechanical recycling, chemical recycling, and emerging recovery technologies are critically compared according to their influence on molecular degradation, property retention, process stability, material quality, and industrial feasibility.

Layer IV—Circularity Performance

The final analytical layer integrates technical performance with environmental, economic, and industrial perspectives. This includes Life Cycle Assessment (LCA), energy consumption, greenhouse gas emissions, material value retention, process scalability, regulatory constraints, and infrastructure availability, thereby evaluating the practical feasibility of closed-loop polymer additive manufacturing.

Rather than treating these analytical layers as independent stages, the proposed framework systematically investigates the causal relationships existing between them. Material degradation accumulated during manufacturing directly influences recycling efficiency, while recycling-induced molecular modifications determine the long-term environmental and economic viability of circular manufacturing systems. Consequently, circularity is interpreted as the cumulative outcome of successive interactions occurring throughout the complete material lifecycle.

This analytical perspective enables the identification of fundamental scientific bottlenecks that remain largely overlooked when manufacturing processes, degradation mechanisms, recycling technologies, or sustainability assessments are investigated separately. By integrating these complementary dimensions within a single conceptual framework, the review establishes a coherent basis for critically interpreting the current literature while identifying the principal scientific and technological priorities required to enable industrial-scale circular polymer additive manufacturing.

**Figure 6:**
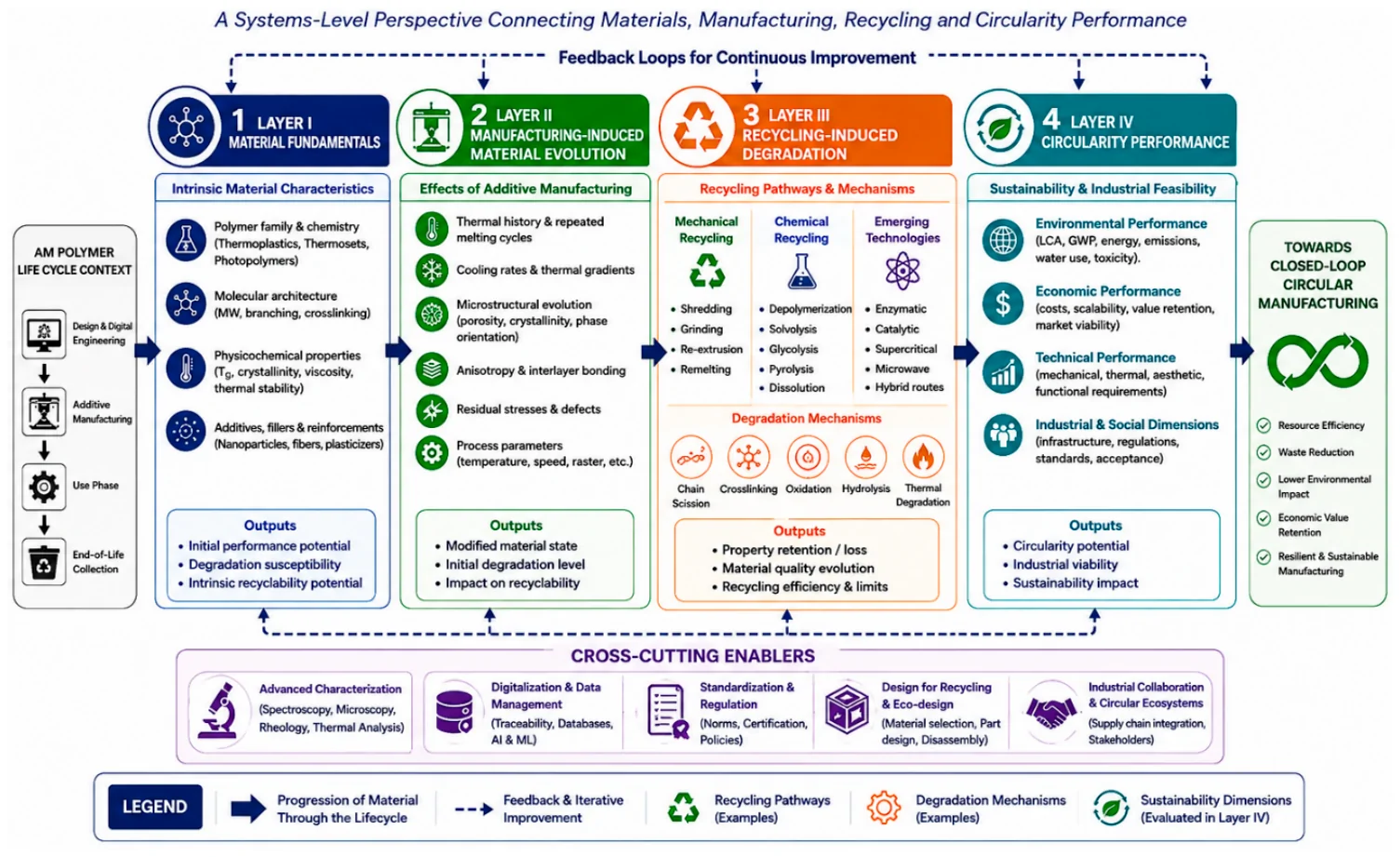
Integrated analytical framework for circular polymer additive manufacturing.

To enable a coherent interpretation of the highly heterogeneous literature, the evidence collected in this review was organized within an integrated analytical framework. As illustrated in Figure 6, polymer circularity is interpreted as a systems-level phenomenon resulting from the interaction between intrinsic material characteristics, manufacturing-induced modifications, recycling pathways, and the resulting environmental, technical, and industrial performance.

The proposed framework provides the conceptual foundation for the critical discussion developed throughout the review, highlighting the causal relationships that govern polymer degradation, recyclability, and circularity across the complete additive manufacturing lifecycle. It also establishes a unified perspective for identifying current technological bottlenecks and future research priorities.

The integrated analytical framework adopted in this review establishes the methodological basis of the proposed ICPAM perspective. Accordingly, the following sections progressively examine the scientific foundations of circular polymer additive manufacturing, beginning with the characteristics of polymer feedstocks and additive manufacturing technologies before analysing degradation mechanisms, recycling pathways, and their combined influence on circularity performance.

## 3. Scientific Foundations of Circular Polymer Additive Manufacturing

The transition towards circular polymer additive manufacturing requires a comprehensive understanding of the scientific mechanisms governing material behaviour throughout the entire manufacturing lifecycle. Although additive manufacturing technologies have considerably expanded the design freedom and production flexibility of polymer components, their long-term sustainability depends not only on manufacturing efficiency but also on the ability of materials to retain their functional performance after repeated processing and recycling cycles.

Unlike conventional polymer processing, additive manufacturing subjects materials to highly process-specific thermal, mechanical, and physicochemical histories that significantly influence their subsequent recyclability. Layer-by-layer fabrication, repeated thermal exposure, localized melting and solidification, photopolymerization, and powder reuse introduce degradation phenomena that progressively modify polymer structure, molecular architecture, and processing behaviour. Consequently, the circularity of polymer additive manufacturing cannot be interpreted exclusively from the perspective of recycling technologies but must instead be understood as the cumulative consequence of interactions occurring between material chemistry, manufacturing conditions, degradation mechanisms, and recovery processes.

This chapter establishes the scientific foundation required for the subsequent critical analysis by progressively examining the relationships between polymer feedstocks, additive manufacturing processes, material degradation, recycling pathways, and system-level circularity. Rather than considering these aspects independently, they are discussed as interconnected elements of an integrated lifecycle framework, thereby providing the conceptual basis for understanding the technological barriers that currently limit fully circular polymer additive manufacturing.

### 3.1. Polymer Feedstocks for Additive Manufacturing

The selection of polymer feedstocks represents one of the most influential factors governing the long-term circularity of Additive Manufacturing (AM) systems. While considerable attention has traditionally been devoted to printability, dimensional accuracy, and mechanical performance, increasing evidence demonstrates that the intrinsic characteristics of polymeric materials largely determine their ability to withstand repeated manufacturing and recycling cycles without experiencing irreversible degradation. Consequently, the circular economy potential of polymer AM should be considered during material selection rather than being addressed solely at the end-of-life stage.

From a materials science perspective, the recyclability of polymers is fundamentally controlled by their molecular architecture and thermal behaviour. Chain structure, molecular weight distribution, crystallinity, glass transition temperature, melting characteristics, chemical stability, and susceptibility to thermo-oxidative or hydrolytic degradation collectively influence the extent to which a material can preserve its functional properties after successive processing cycles. These intrinsic characteristics determine not only the processing window during additive manufacturing but also the mechanisms through which degradation accumulates during service and recycling [17,18].

Thermoplastic polymers dominate current polymer additive manufacturing because their reversible melting behaviour enables repeated thermal processing and, consequently, facilitates mechanical recycling. Materials such as polylactic acid (PLA), acrylonitrile butadiene styrene (ABS), polyethylene terephthalate glycol (PETG), polyamide (PA), polypropylene (PP), polycarbonate (PC), and high-performance engineering polymers including polyether ether ketone (PEEK) exhibit distinct combinations of mechanical performance, thermal stability, chemical resistance, and recyclability. However, these advantages are accompanied by different degradation pathways, making their long-term circularity strongly material-dependent.

For example, PLA has attracted widespread attention due to its renewable origin and industrial compostability under controlled conditions. Nevertheless, repeated thermal processing frequently induces chain scission, molecular weight reduction, and progressive deterioration of mechanical performance, thereby limiting the number of effective recycling cycles without the incorporation of stabilizers or virgin material. ABS generally exhibits greater thermal robustness but remains susceptible to thermo-oxidative degradation, while PETG offers improved processing stability owing to its lower crystallinity and favourable melting behaviour. Polyamides provide excellent mechanical performance but readily absorb moisture, making hydrolytic degradation a critical factor during repeated processing. In contrast, high-performance polymers such as PEEK retain superior thermal and mechanical stability, although their elevated processing temperatures significantly increase energy demand and manufacturing complexity.

Beyond the polymer matrix itself, feedstock quality is also influenced by additives, pigments, reinforcing fibres, compatibilizers, plasticizers, flame retardants, and stabilizing agents. Although these constituents are frequently incorporated to enhance printability or functional performance, they may simultaneously alter degradation kinetics, recycling efficiency, material compatibility, and the physicochemical properties of recycled feedstocks [19]. Consequently, identical polymer families may exhibit substantially different recycling behaviour depending on their specific formulation and manufacturing history.

Another critical aspect concerns the feedstock format employed by different additive manufacturing technologies. Filaments, powders, pellets, liquid photopolymers, and composite feedstocks experience distinct thermal and environmental histories during processing, resulting in different degradation mechanisms before recycling even begins [20]. Powder reuse in powder bed fusion systems, repeated filament extrusion in material extrusion technologies, and irreversible photopolymer crosslinking in vat photopolymerization processes illustrate how manufacturing technology directly conditions the subsequent recyclability of the material [21,22]. Therefore, feedstock selection and processing technology should not be considered independently but rather as coupled variables governing the evolution of material properties throughout the additive manufacturing lifecycle [23].

To facilitate the comparative interpretation of the principal polymer families employed in additive manufacturing, Figure 5 summarizes their relative circularity potential by integrating intrinsic material stability, recyclability, industrial maturity, and the main limitations affecting long-term lifecycle performance. This systems-level comparison highlights that circularity is determined by the combined influence of polymer chemistry, degradation behaviour, and industrial implementation rather than by material composition alone.

**Figure 7:**
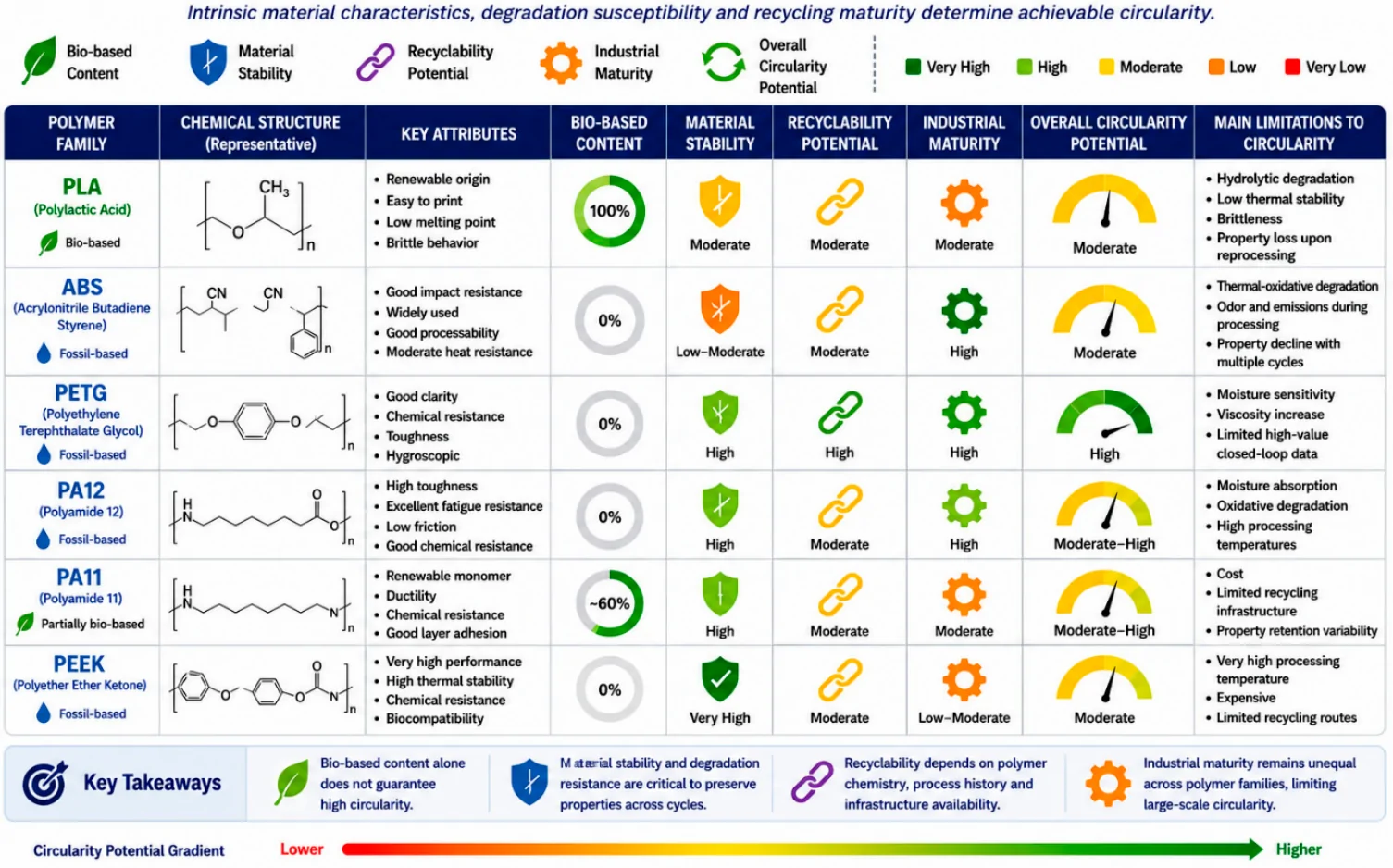
Comparative assessment of polymer families according to their circularity potential in additive manufacturing.

As illustrated in Figure 7, no single polymer simultaneously maximizes material stability, recyclability, and industrial applicability. Instead, the achievable level of circularity results from balancing intrinsic material properties with degradation resistance, manufacturing history, and the availability of suitable recovery infrastructures. This comparative perspective provides the scientific basis for understanding how processing conditions further influence polymer evolution, as discussed in the following section on polymer–process interactions.

Taken together, the available evidence indicates that polymer feedstocks should be evaluated not only according to their processing performance or mechanical properties but also according to their capacity to preserve molecular integrity during repeated manufacturing and recycling operations. From this perspective, circularity emerges as an intrinsic consequence of the interaction between polymer chemistry, feedstock formulation, and processing history rather than as an isolated outcome of recycling technologies. Understanding these material-dependent behaviours provides the scientific basis for analysing how additive manufacturing processes further modify polymer structure, as discussed in the following section.

### 3.2. Polymer–Process Interactions in Additive Manufacturing

The performance and circularity potential of polymer feedstocks are not determined solely by their intrinsic material properties but are also profoundly influenced by the processing conditions imposed during additive manufacturing. Throughout layer-by-layer fabrication, polymers are subjected to complex thermal, mechanical, and physicochemical histories that progressively alter their molecular structure, microstructure, and functional behaviour. Consequently, additive manufacturing should not be regarded as a passive shaping technology but rather as an active stage in the lifecycle during which material evolution begins.

Each additive manufacturing technology generates a distinct processing environment that governs the nature and magnitude of these material transformations [24,25]. Material extrusion processes, such as Fused Deposition Modeling (FDM) and Fused Filament Fabrication (FFF), repeatedly expose polymers to melting, extrusion, deposition, and solidification cycles [26]. Successive thermal exposures may promote chain scission, thermo-oxidative degradation, viscosity reduction, and gradual molecular weight loss, particularly when recycled feedstocks are processed multiple times. Simultaneously, cooling conditions and interlayer diffusion strongly influence interfacial bonding, residual stresses, porosity, and anisotropic mechanical behaviour.

Powder-based technologies, including Selective Laser Sintering (SLS), introduce a different set of process–material interactions. During fabrication, polymer powders remain exposed to elevated temperatures for extended periods while only localized regions undergo complete melting. This prolonged thermal exposure accelerates ageing phenomena, oxidation, changes in molecular weight distribution, and variations in powder flowability. As unused powder is frequently recycled in subsequent production cycles, progressive degradation may accumulate even before the material is incorporated into a printed component, directly affecting dimensional accuracy, mechanical performance, and process repeatability.

Vat photopolymerization processes, including Stereolithography (SLA) and Digital Light Processing (DLP), exhibit fundamentally different material evolution mechanisms [27,28]. Rather than relying on reversible melting, these technologies employ photochemical polymerization reactions that generate highly crosslinked polymer networks. Although excellent dimensional precision and surface quality can be achieved, the irreversible nature of crosslink formation significantly limits conventional mechanical recycling. Furthermore, incomplete polymerization, ultraviolet ageing, and post-curing reactions may continue modifying material properties after fabrication, influencing long-term stability and end-of-life recovery options.

Independent of the specific technology employed, process parameters exert a decisive influence on material evolution. Extrusion temperature, laser power, scanning speed, layer thickness, exposure time, cooling rate, build orientation, chamber temperature, and environmental conditions collectively determine the thermal history experienced by the polymer. These variables control not only print quality and dimensional accuracy but also the accumulation of molecular damage, residual stresses, crystallinity evolution, pore formation, and interlayer adhesion. Consequently, identical polymers may exhibit substantially different degradation behaviour and recyclability depending on the manufacturing conditions under which they were processed [29,30,31].

An additional complexity arises from the cumulative nature of process-induced degradation. Unlike conventional polymer manufacturing, additive manufacturing frequently involves multiple thermal histories before the component reaches its service life, including filament extrusion or powder production, feedstock conditioning, layer-by-layer fabrication, post-processing, and, eventually, recycling. Each processing stage contributes incrementally to molecular degradation, making the final material condition the result of the entire manufacturing history rather than a single fabrication step [32,33]. This cumulative evolution represents one of the principal scientific challenges in establishing reliable closed-loop recycling strategies for polymer additive manufacturing [34].

The interaction between material characteristics and manufacturing conditions therefore constitutes a critical determinant of circularity performance. Process-induced modifications directly influence subsequent degradation mechanisms, property retention during recycling, and the long-term technical and environmental viability of circular manufacturing systems [35]. Understanding these interactions is therefore essential for interpreting the degradation phenomena discussed in the following section, where the fundamental mechanisms responsible for material deterioration throughout the additive manufacturing lifecycle are critically examined.

The interaction between polymer chemistry and additive manufacturing processes determines not only the initial performance of printed components but also their long-term degradation behaviour and recovery potential. To summarize these coupled relationships, Figure 6 provides a comparative overview linking representative polymer families with their typical processing routes, dominant degradation mechanisms, and the principal limitations affecting subsequent recycling operations.

**Figure 8:**
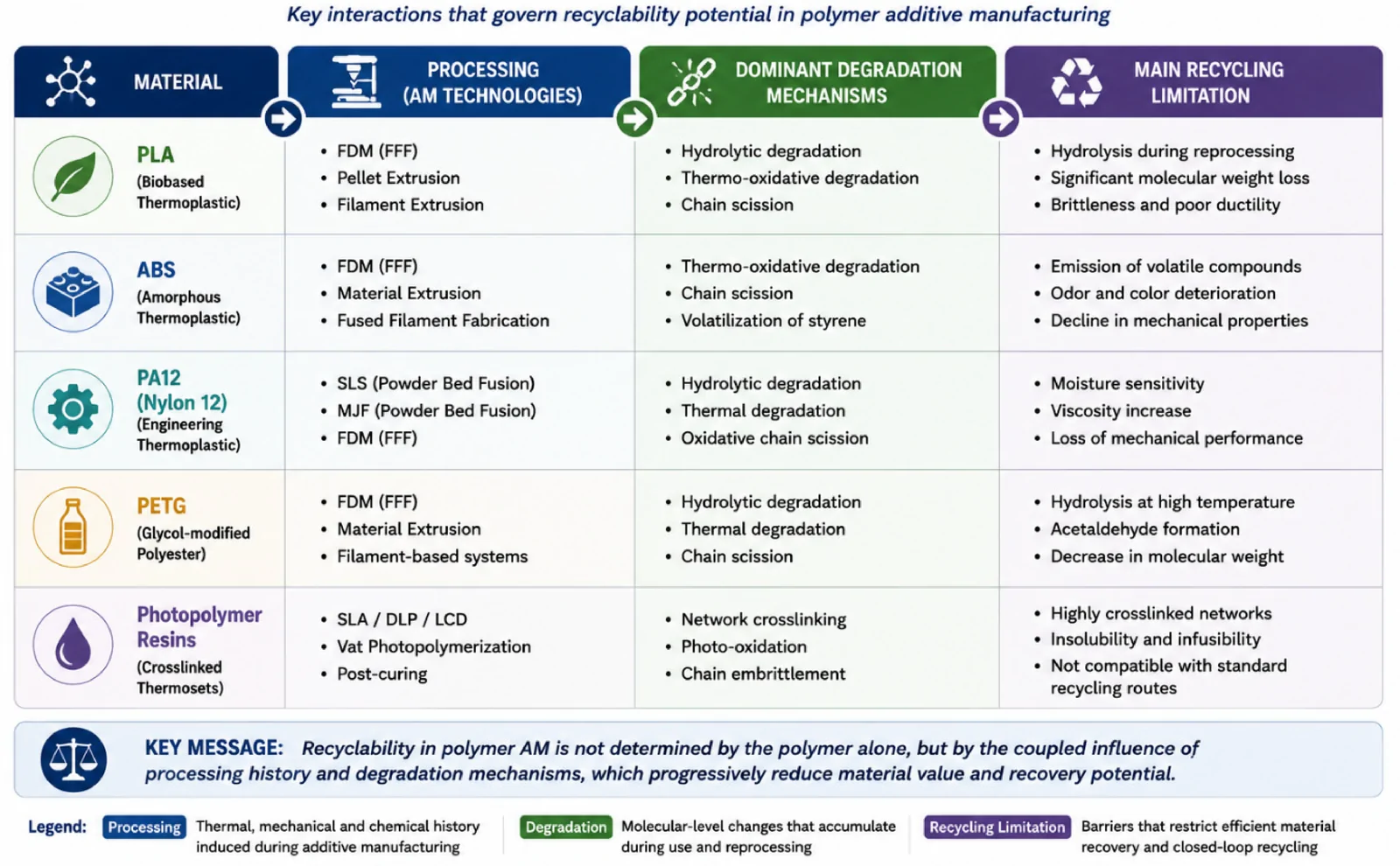
Comparative relationships between polymer type, processing history, degradation mechanisms, and recycling limitations.

As illustrated in Figure 8, the recyclability of polymer feedstocks is governed by the combined effects of material selection and processing history rather than by intrinsic polymer properties alone. This integrated perspective demonstrates that manufacturing-induced degradation progressively constrains recovery potential, providing the scientific basis for the lifecycle degradation mechanisms analysed in the following section.

### 3.3. Degradation Mechanisms Across the Additive Manufacturing Lifecycle

Material degradation represents one of the principal scientific barriers to achieving effective circularity in polymer additive manufacturing. Although recycling technologies are frequently regarded as the primary determinant of material recovery, the long-term performance of recycled polymers is fundamentally governed by degradation phenomena that progressively accumulate throughout the entire additive manufacturing lifecycle. Rather than occurring as isolated events, these mechanisms originate during feedstock production, evolve throughout manufacturing and service, and continue during successive recycling operations, ultimately determining the functional lifespan of polymer materials.

At the molecular level, degradation primarily involves irreversible modifications of polymer chain architecture. Thermal exposure, oxidative reactions, hydrolysis, ultraviolet radiation, mechanical stresses, and environmental ageing promote chain scission, crosslinking, oxidation, depolymerization, and molecular rearrangement. These physicochemical transformations alter molecular weight distribution, crystallinity, viscosity, thermal stability, and mechanical performance, progressively reducing the ability of polymers to withstand repeated processing cycles while maintaining acceptable engineering properties [36].

Thermal degradation constitutes one of the most pervasive mechanisms in polymer additive manufacturing. During extrusion-based processes, polymers repeatedly experience melting and solidification cycles that may exceed their optimal processing window, particularly when recycled feedstocks are reprocessed multiple times. Elevated temperatures accelerate molecular chain scission and thermo-oxidative reactions, reducing melt viscosity and compromising interlayer bonding, dimensional stability, and mechanical strength. The cumulative nature of thermal degradation explains why material performance frequently deteriorates even when each individual processing cycle remains within nominal operating conditions [37,38].

Oxidative degradation further intensifies these effects through reactions between polymer chains and atmospheric oxygen during processing, storage, and service. Oxidation promotes the formation of oxygen-containing functional groups, increasing material brittleness and accelerating subsequent chain fragmentation. The severity of oxidative degradation depends on polymer chemistry, processing temperature, residence time, and environmental exposure, making oxygen availability an important variable in determining long-term recyclability.

Hydrolytic degradation represents another critical pathway, particularly for moisture-sensitive polymers such as polyamides and polyesters. Water molecules diffuse into the polymer matrix and cleave susceptible chemical bonds, reducing molecular weight and compromising both mechanical integrity and processing behaviour. Moisture absorbed during storage or feedstock handling may therefore initiate degradation before manufacturing even begins, highlighting the importance of feedstock conditioning as part of circular manufacturing strategies.

Photochemical degradation becomes especially relevant in vat photopolymerization technologies and during prolonged environmental exposure. Ultraviolet radiation initiates bond cleavage and photo-oxidative reactions that progressively alter polymer network architecture, surface chemistry, and colour stability. In photopolymer systems, continued post-curing and irreversible crosslink formation further restrict the possibility of conventional mechanical recycling, making end-of-life recovery substantially more challenging than for thermoplastic materials.

Mechanical degradation also contributes to cumulative material deterioration. Shear stresses generated during extrusion, filament production, pelletization, grinding, shredding, and repeated recycling operations progressively damage polymer chains while modifying particle morphology and rheological behaviour. Although these mechanical effects are frequently less pronounced than thermal or oxidative degradation, their repeated occurrence across multiple processing cycles contributes significantly to the gradual loss of material performance [39,40].

Importantly, degradation mechanisms rarely occur independently. Thermal exposure frequently accelerates oxidation, oxidation increases susceptibility to mechanical failure, hydrolysis reduces thermal stability, and photochemical ageing promotes further oxidative reactions. These coupled degradation processes generate synergistic effects that are considerably more severe than the individual mechanisms considered separately [41,42]. Consequently, predicting polymer durability requires a holistic understanding of the interactions between multiple degradation pathways operating simultaneously throughout the material lifecycle.

From the perspective of circular manufacturing, degradation should therefore be interpreted as a cumulative lifecycle phenomenon rather than as a consequence of individual recycling operations. Feedstock production, additive manufacturing, post-processing, service conditions, waste collection, material recovery, and successive recycling cycles each contribute incrementally to molecular deterioration. The residual performance of recycled polymers consequently reflects the entire processing history experienced by the material rather than the efficiency of the recycling technology alone [43,44].

**Figure 9:**
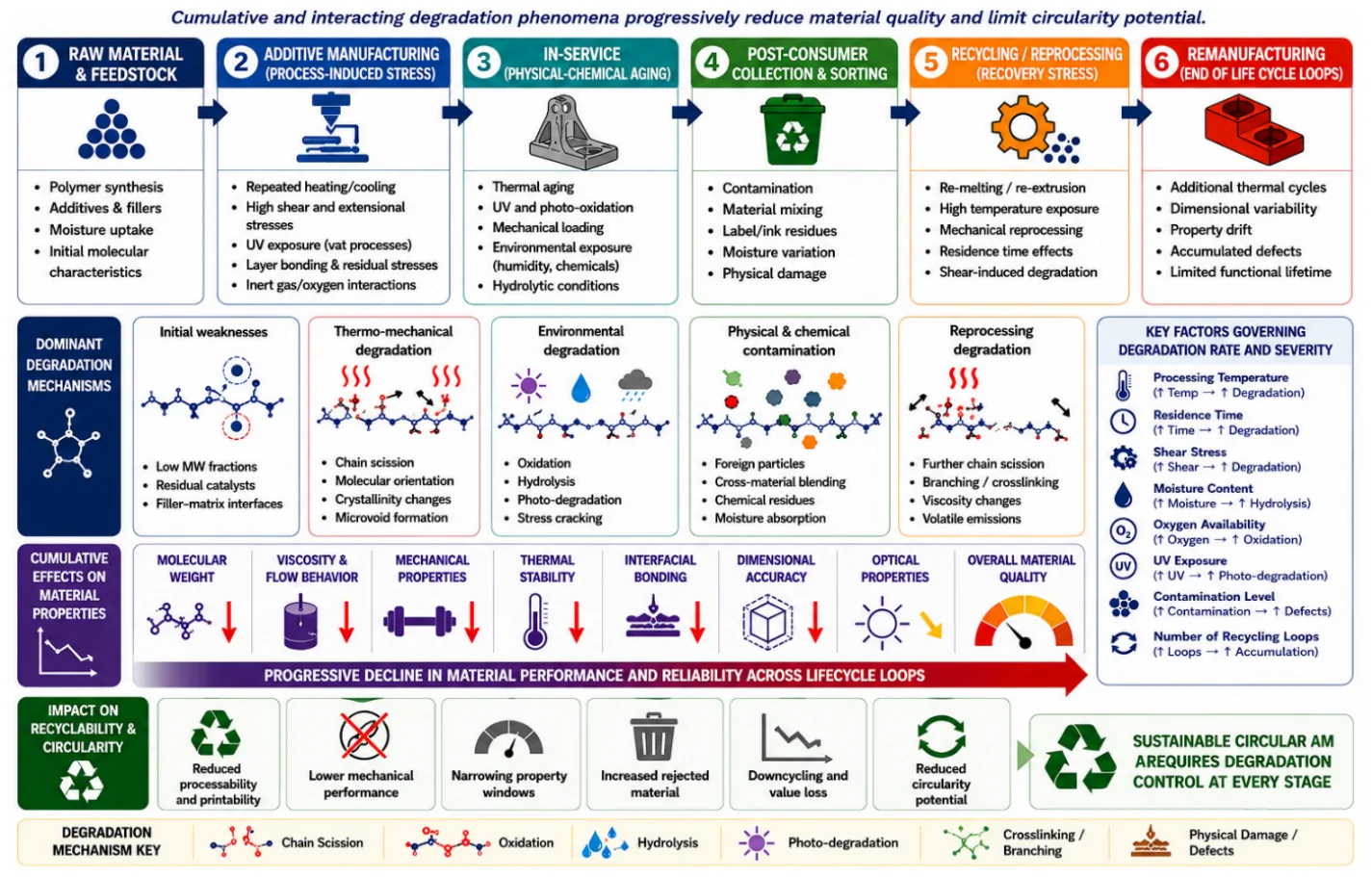
Lifecycle degradation mechanisms governing polymer circularity in additive manufacturing.

The degradation mechanisms discussed throughout this section should not be interpreted as isolated phenomena but as cumulative and interacting processes that progressively determine the long-term recyclability of polymer feedstocks. As illustrated in Figure 9, thermal, oxidative, hydrolytic, photochemical, and mechanical degradation accumulate throughout successive lifecycle stages, progressively reducing material quality, property retention, and circularity potential.

Figure 9 emphasizes that material degradation is initiated long before end-of-life recovery and continuously evolves throughout manufacturing, service, and recycling. This lifecycle perspective demonstrates that preserving polymer circularity depends not only on efficient recycling technologies but also on minimizing cumulative degradation during every stage of the additive manufacturing process, thereby providing the scientific foundation for the evaluation of advanced recycling pathways presented in the following section [45].

Understanding the mechanisms governing polymer degradation is therefore essential for designing effective circular manufacturing strategies. It enables the identification of processing conditions that minimize irreversible molecular damage, supports the development of stabilization and material recovery techniques, and provides the scientific basis for selecting recycling pathways capable of maximizing property retention throughout successive manufacturing cycles. These considerations naturally lead to the evaluation of the recycling technologies currently available for polymer additive manufacturing, which are critically analysed in the following section.

### 3.4. Recycling Pathways and Material Recovery

The successful implementation of circular polymer additive manufacturing ultimately depends on the effectiveness of the recycling pathways adopted to recover material functionality after degradation has occurred. However, recycling should not be interpreted simply as a waste management strategy. Instead, it represents an engineering process aimed at preserving molecular integrity, restoring material performance, and maximizing resource efficiency throughout successive manufacturing cycles. The selection of an appropriate recycling pathway therefore plays a decisive role in determining whether polymer materials can realistically remain within closed-loop manufacturing systems.

To translate the ICPAM architecture into an operational decision-support process, circular strategy selection must extend beyond the simultaneous consideration of multiple performance criteria. As illustrated in Figure 8, the proposed methodology follows a sequential pathway in which the current material, manufacturing, degradation, lifecycle-performance, and ICPAM maturity states are first characterized. Technically, regulatorily, infrastructurally, or application-incompatible alternatives are subsequently excluded through feasibility screening, after which the remaining circular strategies are evaluated against technical, environmental, economic, circularity, and industrial criteria. Normalization and weighting place these heterogeneous criteria on a comparable decision basis, enabling the feasible alternatives to be ranked using a context-appropriate MCDM method and subsequently tested for robustness before final selection.

**Figure 10:**
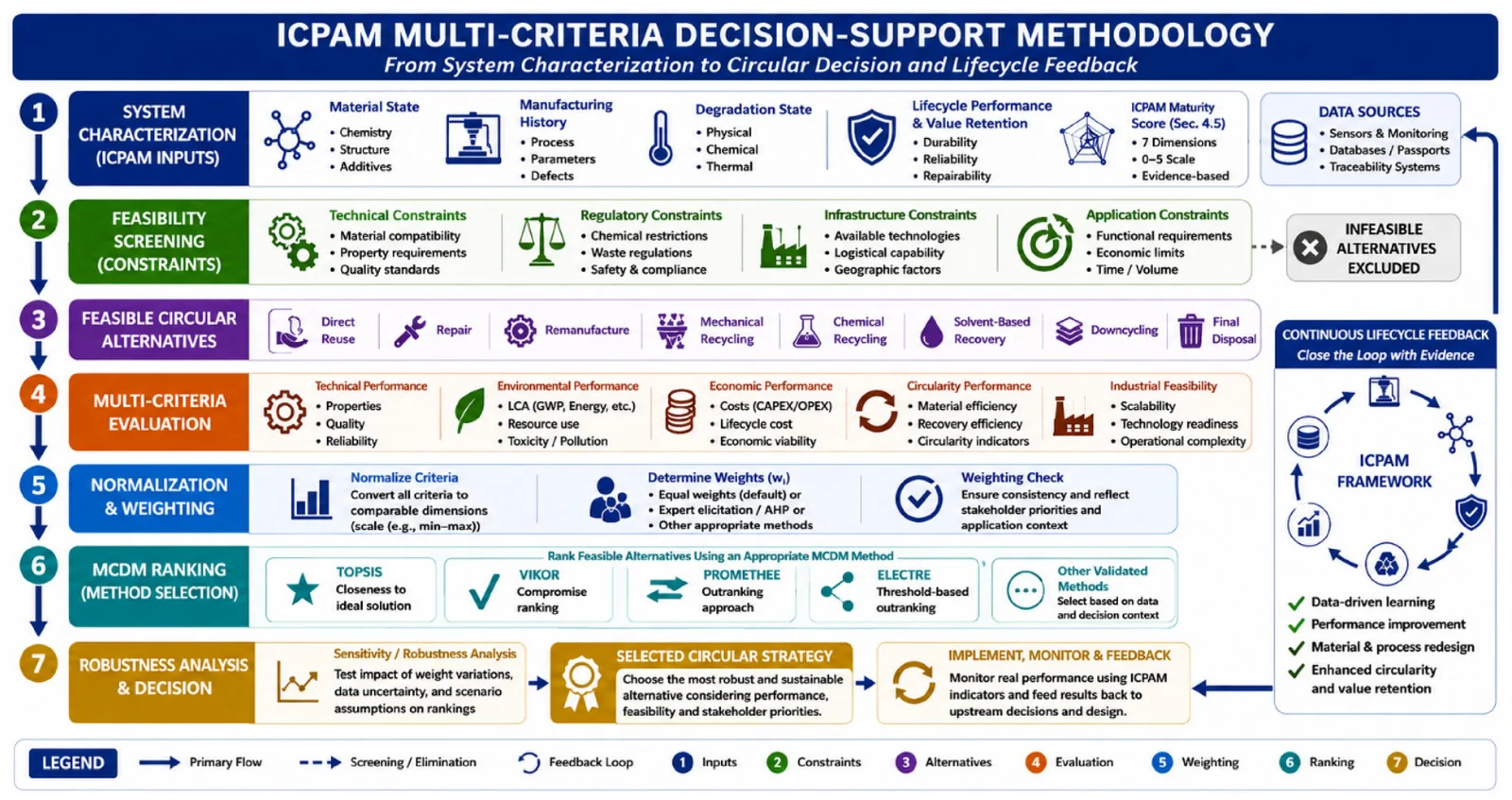
ICPAM multi-criteria decision-support methodology integrating system characterization, feasibility screening, circular alternatives, lifecycle criteria, normalization and weighting, MCDM ranking, robustness analysis, strategy selection, and continuous lifecycle feedback.

Figure 8 therefore clarifies that ICPAM does not prescribe a circular strategy directly from a predefined set of criteria; instead, it structures the evidence required to progressively eliminate infeasible alternatives and identify the most appropriate solution for a specific material, product, and industrial context. The distinction between feasibility screening and multi-criteria ranking is particularly important, since technically or regulatorily unacceptable options should not be compensated by favourable performance in other criteria. For the remaining alternatives, the framework is deliberately MCDM-compatible rather than method-specific: equal weighting may provide a neutral baseline, whereas expert elicitation or AHP may support context-dependent weighting, and established approaches such as TOPSIS, VIKOR, PROMETHEE, or ELECTRE may subsequently be selected according to the data structure and decision problem. Sensitivity analysis then evaluates ranking robustness before implementation. Finally, measured lifecycle performance is returned to the ICPAM architecture, converting decision-making from a one-time selection exercise into a continuously learning closed-loop process. Accordingly, Figure 2 identifies where decision support occurs across the lifecycle, whereas Figure 10 details how a specific circular-strategy decision is operationalized once a decision gate is reached.

Importantly, ICPAM is not intended to support decision-making at a single isolated lifecycle stage. Instead, it operates through multiple decision gates distributed across the polymer AM lifecycle. At the design and material-selection stage, it supports decisions concerning polymer chemistry, formulation, durability, repairability, and recovery compatibility. During manufacturing, it assists in selecting and adapting processing conditions according to material state, thermal history, degradation risk, and quality requirements. During use and service, lifecycle evidence supports decisions concerning continued use, maintenance, repair, reuse, or remanufacturing. Finally, when functional value can no longer be adequately retained at product level, ICPAM supports the selection of the most appropriate recovery pathway through the multi-criteria methodology illustrated in Figure 8. Circularity, sustainability, and operational performance measured after implementation are subsequently returned to upstream design and manufacturing decisions, establishing a continuous lifecycle feedback mechanism.

Mechanical recycling remains the most widely implemented recovery strategy for polymer additive manufacturing due to its technological maturity, relatively low processing cost, and compatibility with most thermoplastic feedstocks [46]. The process generally involves collection, sorting, cleaning, shredding, re-melting, and re-extrusion of polymer waste into new feedstock forms such as filaments, pellets, or powders. Although this approach enables straightforward material recovery with comparatively low environmental impact, repeated thermal and mechanical processing progressively accelerates molecular degradation, leading to reductions in molecular weight, viscosity, mechanical strength, and dimensional stability. Consequently, mechanical recycling is frequently limited by the gradual accumulation of irreversible material damage, requiring the incorporation of virgin polymers or stabilizing additives to maintain acceptable processing performance.

Chemical recycling offers an alternative route by targeting the molecular structure of degraded polymers rather than simply reprocessing the existing material [47]. Through depolymerization, solvolysis, pyrolysis, glycolysis, hydrolysis, or related chemical conversion processes, polymers can be transformed into monomers or intermediate chemical products suitable for subsequent repolymerization. This strategy has the potential to restore material quality more effectively than conventional mechanical recycling while enabling the treatment of more complex or contaminated waste streams. Nevertheless, chemical recycling generally requires higher energy inputs, more sophisticated processing infrastructure, greater economic investment, and careful control of reaction conditions, which currently limits its widespread industrial implementation.

Emerging recycling approaches seek to overcome the limitations associated with conventional recovery technologies by combining material engineering with advanced processing strategies. Solvent-based purification methods, dissolution–reprecipitation techniques, reactive extrusion, dynamic covalent polymer systems, vitrimer chemistry, self-healing polymers, and bio-based recyclable thermosets have demonstrated promising potential for extending material lifespan while improving property retention after repeated processing. Although many of these technologies remain at relatively low technology readiness levels, they illustrate the growing transition from waste treatment towards material regeneration and molecular design for circularity.

Material recovery performance is strongly influenced by feedstock quality before recycling begins [48,49]. Contamination, moisture uptake, oxidation, filler dispersion, pigment composition, and the presence of multiple polymer families frequently reduce recycling efficiency and complicate material separation. Furthermore, additive manufacturing waste exhibits considerable heterogeneity, including failed prints, support structures, unused powders, purge materials, and post-processing residues, each presenting distinct degradation histories and recovery requirements [50]. Consequently, effective recycling strategies must consider not only the selected recovery technology but also the characteristics of the incoming waste stream [51].

An equally important consideration concerns the compatibility between recycling technologies and the various additive manufacturing processes. Thermoplastic materials processed through material extrusion generally exhibit the highest potential for repeated mechanical recycling due to their reversible melting behaviour. In contrast, powder reuse strategies employed in powder bed fusion require careful monitoring of powder ageing and thermal history to maintain process consistency [52]. Vat photopolymerization systems present substantially greater challenges because irreversible crosslinking severely restricts conventional material recovery, often requiring chemical conversion or energy recovery instead of closed-loop recycling. These technological differences demonstrate that recycling pathways cannot be universally applied across all polymer additive manufacturing processes.

From an industrial perspective, the effectiveness of material recovery should not be evaluated solely according to recycling efficiency or property retention. Equally important are energy demand, greenhouse gas emissions, economic viability, process scalability, logistics, regulatory compliance, and integration within existing manufacturing infrastructures [53]. A recycling technology capable of preserving material properties may nevertheless prove unsuitable for industrial implementation if excessive costs, limited throughput, or inadequate collection systems compromise its practical feasibility. Consequently, technical performance and industrial applicability must be considered simultaneously when assessing circular manufacturing strategies.

Overall, the current literature indicates that no single recycling pathway can adequately address the diversity of polymer materials, additive manufacturing technologies, and degradation histories encountered in industrial practice. Instead, effective circular manufacturing requires an integrated approach in which material selection, process optimization, degradation mitigation, and recycling technology are jointly considered as interconnected elements of a unified lifecycle strategy [54]. This systems’ perspective provides the foundation for understanding the broader technological, environmental, and industrial barriers that continue to limit the implementation of truly circular polymer additive manufacturing, which are discussed in the following section.

### 3.5. Circularity Challenges and System-Level Constraints

Despite the significant advances achieved in polymer additive manufacturing and recycling technologies, the transition from linear production models to fully circular manufacturing systems remains constrained by a series of interconnected scientific, technological, economic, and organizational barriers. Current research increasingly recognizes that circularity cannot be achieved solely through improvements in individual recycling processes. Instead, it depends on the coordinated optimization of the entire material lifecycle, from feedstock design and manufacturing to product use, collection, recovery, and reintegration into new production cycles.

One of the principal challenges arises from the progressive loss of material quality throughout successive manufacturing and recycling operations. Even under carefully controlled processing conditions, cumulative degradation gradually reduces molecular weight, alters rheological behaviour, modifies crystallinity, and deteriorates mechanical performance. Although stabilization strategies and virgin material blending can partially compensate for these effects, they rarely eliminate degradation completely, thereby limiting the number of effective closed-loop recycling cycles that can be achieved for many polymer systems [55,56,57].

Material heterogeneity represents another major obstacle to industrial circularity. Polymer waste generated by additive manufacturing encompasses a wide variety of feedstocks, including different polymer families, reinforced composites, coloured materials, support structures, failed prints, contaminated powders, and post-processing residues. The coexistence of multiple material formulations significantly complicates sorting, identification, compatibility assessment, and recycling operations, reducing both recovery efficiency and the quality of recycled feedstocks [58,59]. These challenges become even more pronounced as multi-material and functionally graded additive manufacturing technologies continue to evolve [60,61,62].

The absence of standardized recycling protocols further limits the scalability of circular manufacturing. Differences in feedstock specifications, processing parameters, material characterization methodologies, and quality control procedures hinder direct comparison between studies and reduce confidence in recycled material performance [63,64,65]. Although international standardization efforts are progressing, harmonized protocols specifically addressing recycled feedstocks for additive manufacturing remain relatively limited, restricting industrial implementation and certification [66,67,68].

Economic considerations also play a decisive role in determining the feasibility of circular manufacturing systems. In many industrial sectors, the cost of collecting, sorting, characterizing, and reprocessing polymer waste may exceed the economic value of the recovered material, particularly when virgin polymers remain readily available at competitive prices [69,70,71,72,73,74,75,76,77]. Furthermore, investments required for advanced recycling infrastructure, quality assurance, and process monitoring frequently represent substantial barriers for small and medium-sized enterprises, delaying the widespread adoption of closed-loop manufacturing strategies [78,79,80,81,82,83,84,85,86,87,88].

Environmental performance presents an equally complex challenge. Although recycling generally reduces material consumption and landfill disposal, its overall sustainability depends on the balance between resource recovery, energy demand, transportation requirements, emissions, and process efficiency. Consequently, recycling cannot automatically be assumed to provide environmental benefits under all circumstances [89,90,91,92,93,94,95,96]. Instead, comprehensive lifecycle assessments are required to determine whether a particular recovery pathway genuinely improves environmental performance when evaluated across the complete manufacturing system [97,98,99,100,101]. Accordingly, lifecycle sustainability should not be inferred from energy consumption alone but evaluated through the combined consideration of environmental burdens, material conservation and value retention, end-of-life recovery or disposal pathways, and, where appropriate, lifecycle economic performance.

Another critical limitation concerns the insufficient integration of digital technologies within circular manufacturing strategies. Despite the rapid development of Industry 4.0 concepts, Digital Twins, artificial intelligence, blockchain, smart sensing, and cloud-based manufacturing platforms, their application to polymer circularity remains relatively fragmented. Digital traceability of material history, predictive degradation modelling, intelligent sorting systems, and real-time quality monitoring offer considerable opportunities to improve recycling efficiency and support data-driven decision-making [102,103,104,105]. However, these technologies have not yet been systematically integrated into most industrial recycling workflows.

Ultimately, the available evidence demonstrates that the principal barriers to circular polymer additive manufacturing extend well beyond material science alone. Circularity emerges from the interaction between material behaviour, process engineering, recycling technologies, digital infrastructure, economic viability, environmental performance, regulatory frameworks, and supply chain organization. Failure in any one of these interconnected dimensions may compromise the effectiveness of the entire circular system, regardless of advances achieved in the remaining components.

Therefore, future progress will require a transition from technology-centred optimization towards systems-oriented engineering strategies capable of simultaneously addressing material durability, process efficiency, resource recovery, lifecycle sustainability, and digital integration. Such an approach provides the conceptual foundation for the synthesis presented in the following section, where the principal scientific insights emerging from the reviewed literature are consolidated into an integrated perspective on circular polymer additive manufacturing.

### 3.6. Synthesis: From Polymer Design to Circular Manufacturing

The analysis presented throughout this chapter demonstrates that circular polymer additive manufacturing cannot be understood through isolated evaluations of material properties, manufacturing technologies, or recycling processes. Instead, circularity emerges from the continuous interaction between polymer chemistry, process-induced material evolution, degradation mechanisms, recovery technologies, and broader industrial and environmental constraints. Consequently, the performance of recycled materials reflects the cumulative effects of the entire manufacturing lifecycle rather than the efficiency of any single processing stage.

This systems-level interpretation highlights a fundamental shift in the way circular manufacturing should be approached. Traditionally, recycling has been considered the primary strategy for improving sustainability in polymer-based manufacturing systems. However, the evidence reviewed indicates that the effectiveness of recycling is largely predetermined by decisions taken during material selection, feedstock formulation, process optimization, and product design. In other words, the capacity to recover material value at the end of life is strongly influenced by engineering choices made long before recycling is initiated [106,107].

Accordingly, the transition towards circular additive manufacturing requires the adoption of design-for-circularity principles that explicitly incorporate recyclability as a material design objective. Such an approach extends beyond conventional design-for-manufacturing methodologies by simultaneously considering molecular stability, processing conditions, degradation resistance, material traceability, recovery potential, and lifecycle sustainability [108,109]. Integrating these aspects during the early stages of product development offers significant opportunities to enhance both technical performance and long-term resource efficiency [110].

From a Life Cycle Engineering (LCE) perspective, however, circularity must extend beyond material design, manufacturing, and end-of-life recycling. The complete product lifecycle also encompasses distribution, use and service, maintenance and repair, reliability and maintainability, reuse or remanufacturing, reverse logistics, and, when further value recovery is technically or environmentally unjustified, final disposal. These stages are interconnected because decisions made during material and product design influence not only manufacturing performance and recyclability but also component durability, service life, reparability, maintenance requirements, and the feasibility of subsequent recovery strategies. Accordingly, circular polymer additive manufacturing should seek to maximize functional and material value across the entire lifecycle rather than optimize recycling as an isolated end-of-life operation [111,112,113].

The reviewed literature also reveals that technological progress alone will not be sufficient to achieve industrial-scale circularity [114,115]. Although considerable advances have been made in polymer development, additive manufacturing processes, and recycling technologies, these innovations must be supported by standardized characterization protocols, digital traceability systems, intelligent process monitoring, robust lifecycle assessment methodologies, and economically viable collection and recovery infrastructures [116,117]. Only through the coordinated development of these complementary elements can truly closed-loop manufacturing systems be established.

From a broader scientific perspective, circular polymer additive manufacturing should therefore be regarded as a multidisciplinary engineering challenge requiring the integration of materials science, manufacturing engineering, polymer chemistry, environmental assessment, digital technologies, industrial management, and circular economy principles [118,119]. The interaction between these disciplines ultimately determines the capacity of polymer materials to retain value throughout successive manufacturing and recovery cycles.

The integrated analytical framework proposed in this review provides a unified perspective for interpreting these complex interactions, moving beyond technology-centred analyses towards a lifecycle-oriented understanding of circular manufacturing [120]. By establishing explicit relationships between material design, process evolution, degradation behaviour, recycling performance, and system-level sustainability, the framework offers the scientific foundations collectively explaining the material-level mechanisms that support the proposed ICPAM framework.

This synthesis concludes the scientific foundations of circular polymer additive manufacturing and establishes the conceptual transition to the following chapters, where the reviewed evidence is critically analysed in greater detail to identify emerging technological trends, industrial implementation strategies, and future research priorities for enabling truly circular polymer additive manufacturing.

## 4. Enabling Circular Polymer Additive Manufacturing

### 4.1. Material Design Strategies for Enhanced Circularity

The transition towards circular polymer additive manufacturing requires a fundamental shift in material development philosophy. Rather than considering recyclability as a downstream waste management issue, contemporary research increasingly recognizes that circularity should be embedded within the initial stages of material design. Consequently, polymer feedstocks are progressively being engineered not only to satisfy manufacturing and mechanical performance requirements but also to preserve molecular integrity, facilitate material recovery, and maintain functional properties throughout successive processing and recycling cycles.

In the context of polymer additive manufacturing, Design for Circularity (DfC) refers to the deliberate integration of circular-economy requirements into material, product, and process design from the earliest engineering stages, with the objective of preserving material functionality and value across multiple lifecycles. Unlike conventional design approaches primarily focused on manufacturability and in-service performance, DfC anticipates material durability, repairability, reuse, recyclability, separation, traceability, and end-of-life recovery as explicit design requirements. At the material level, this involves jointly considering polymer chemistry, molecular architecture, additive formulation, processing behaviour, degradation resistance, and recovery compatibility; at the product and manufacturing levels, it requires avoiding design choices that unnecessarily hinder material separation or high-value recovery. Thus, within the ICPAM framework, DfC shifts circularity from a downstream waste-management intervention to an upstream engineering strategy in which decisions made before manufacturing determine the capacity to retain material value through successive use, recovery, and remanufacturing cycles.

One of the principal strategies for improving circularity involves the development of polymers exhibiting enhanced resistance to thermal, oxidative, and hydrolytic degradation [121]. Increasing molecular stability reduces irreversible chain scission during repeated manufacturing cycles, thereby improving property retention after recycling. The incorporation of thermal stabilizers, antioxidants, ultraviolet absorbers, and chain extenders has demonstrated considerable potential for mitigating molecular degradation while extending the useful lifespan of recycled feedstocks. Nevertheless, the long-term effectiveness of these additives depends on their compatibility with the polymer matrix and their stability during successive processing operations.

Another promising direction concerns the optimization of polymer formulations specifically intended for additive manufacturing. Conventional polymers originally developed for injection moulding or extrusion frequently exhibit limited performance when subjected to the repeated thermal histories characteristic of layer-by-layer fabrication. Consequently, increasing research efforts focus on tailoring molecular weight distribution, melt rheology, crystallization behaviour, and interlayer diffusion characteristics to simultaneously improve printability, structural integrity, and recyclability. These optimized formulations contribute to reducing processing-induced degradation while enhancing the stability of recycled materials.

Dynamic polymer networks have emerged as one of the most promising material classes for future circular manufacturing systems. Unlike conventional thermosetting polymers, which form permanent crosslinked structures, dynamic covalent polymers and vitrimers incorporate reversible chemical bonds capable of rearranging under controlled thermal or chemical stimuli. This reversible network topology enables reshaping, repair, welding, and repeated material recovery without completely sacrificing structural performance. Although their industrial adoption remains limited, these materials illustrate a paradigm shift towards polymers specifically designed for multiple lifecycles rather than single-use applications.

Bio-based and biodegradable polymers have also attracted considerable attention within sustainable additive manufacturing. Materials such as polylactic acid (PLA) demonstrate the potential to reduce dependence on fossil-derived feedstocks while contributing to lower environmental impacts under appropriate end-of-life conditions. However, biodegradability should not be regarded as synonymous with circularity. Industrial compostability frequently requires controlled environmental conditions that differ substantially from conventional waste management infrastructures, and repeated mechanical recycling may still induce progressive molecular degradation. Consequently, the selection of bio-based polymers should consider the complete lifecycle context rather than relying solely on renewable origin or biodegradation potential.

Material circularity is further influenced by the presence of fillers, reinforcing fibres, pigments, flame retardants, and functional additives [122,123]. Although these constituents frequently improve stiffness, strength, wear resistance, thermal stability, or printability, they may simultaneously complicate recycling through phase separation, contamination, or incompatibility between material components [124]. Future material design therefore requires a balanced optimization between functional performance and recyclability, ensuring that multifunctional formulations remain compatible with efficient recovery processes [125].

Recent advances in computational materials engineering provide additional opportunities for accelerating the development of circular polymer systems. Machine learning, molecular modelling, high-throughput material screening, and data-driven materials informatics increasingly enable the prediction of degradation behaviour, processing performance, and recyclability before experimental validation. These digital approaches considerably reduce development time while supporting the identification of polymer formulations specifically optimized for circular manufacturing environments.

Overall, the available evidence indicates that achieving circular polymer additive manufacturing depends fundamentally on engineering materials capable of maintaining functionality across multiple manufacturing and recovery cycles. Rather than optimizing isolated material properties, future polymer development should adopt an integrated lifecycle perspective in which molecular stability, processability, recyclability, environmental performance, and economic feasibility are simultaneously considered [126,127]. This evolution from material design towards lifecycle-oriented engineering establishes the foundation for the process optimization strategies discussed in the following section, where manufacturing conditions are analysed as key variables for preserving material quality throughout circular production systems [128].

### 4.2. Process Optimization for Material Preservation

The development of circular polymer feedstocks alone is insufficient to ensure long-term material sustainability if manufacturing conditions promote premature degradation. Throughout additive manufacturing, polymers are exposed to complex thermal, mechanical, and environmental conditions that progressively influence molecular integrity, microstructural evolution, and functional performance. Consequently, process optimization represents a fundamental strategy for preserving material quality and extending the number of effective manufacturing and recycling cycles that can be achieved within circular production systems.

Unlike conventional manufacturing processes, additive manufacturing subjects polymers to highly localized and repetitive thermal histories. During extrusion, sintering, or photopolymerization, materials experience successive heating and cooling cycles that may accelerate chain scission, oxidation, crystallinity evolution, residual stress formation, and viscosity reduction. Although these phenomena are often discussed individually, they collectively determine the progressive deterioration of recycled feedstocks. Therefore, process optimization should be regarded not only as a means of improving print quality but also as a mechanism for minimizing cumulative molecular degradation [129,130].

Among the numerous processing variables, temperature management remains one of the most influential factors governing material preservation. Extrusion temperature, chamber temperature, laser energy density, exposure time, and cooling rate directly affect polymer stability by controlling the duration and severity of thermal exposure. Processing conditions exceeding the optimal thermal window significantly accelerate degradation reactions, whereas insufficient thermal energy may compromise interlayer bonding and structural integrity. The optimization of thermal profiles therefore requires balancing manufacturing performance with long-term material durability [131].

Residence time constitutes an additional parameter of particular importance. Prolonged exposure of molten polymers to elevated temperatures increases the probability of thermo-oxidative reactions and molecular chain scission, even when nominal processing temperatures remain within recommended limits. Minimizing unnecessary thermal residence through optimized process planning, improved material flow, and efficient production scheduling can substantially reduce degradation while maintaining manufacturing productivity.

Environmental control also contributes significantly to material preservation. Moisture uptake during feedstock storage or processing promotes hydrolytic degradation in hygroscopic polymers, while oxygen availability accelerates oxidation during repeated thermal cycles [132]. Controlled storage conditions, feedstock drying procedures, inert processing atmospheres, and environmental monitoring therefore represent effective strategies for mitigating degradation before and during manufacturing. These relatively simple interventions frequently provide substantial improvements in recycled material quality without requiring major modifications to manufacturing equipment [133].

Process optimization further extends to the selection of build orientation, layer thickness, scanning strategy, deposition path, and energy input. These parameters influence heat accumulation, interlayer diffusion, porosity formation, residual stress development, and cooling behaviour, thereby affecting both component performance and the extent of material degradation [134,135]. Optimized process planning can reduce unnecessary thermal cycling while improving process repeatability and material utilization, contributing simultaneously to product quality and circularity [136].

Increasing attention has also been devoted to real-time process monitoring and adaptive process control. Advanced sensing technologies, infrared thermography, machine vision, acoustic emission monitoring, and data-driven control algorithms enable continuous observation of manufacturing conditions and facilitate dynamic adjustment of process parameters whenever deviations from optimal operating conditions are detected. Such closed-loop control systems not only improve manufacturing consistency but also reduce the accumulation of irreversible molecular damage, thereby extending the useful life of polymer feedstocks [137,138].

Digital manufacturing technologies are expected to further strengthen process optimization in future circular production systems. Artificial intelligence, machine learning, Digital Twins, and predictive modelling increasingly enable the simulation of thermal histories, degradation evolution, and process stability before physical manufacturing begins. By integrating material behaviour models with real-time manufacturing data, these digital tools offer the possibility of predicting degradation trajectories and optimizing process parameters to maximize material preservation throughout successive production and recycling cycles [139,140].

Overall, the available evidence demonstrates that process optimization should be interpreted as a central component of circular manufacturing rather than merely as a strategy for improving production efficiency. Preserving molecular integrity during fabrication directly increases the quality of recycled feedstocks, extends material lifespan, and reduces the demand for virgin polymer replacement [141,142,143]. Consequently, manufacturing optimization establishes a critical link between material design and recycling performance, reinforcing the systems-oriented perspective adopted throughout this review. This integrated approach naturally leads to the examination of advanced recycling technologies, which seek to recover the remaining material value after degradation has inevitably occurred.

### 4.3. Recovering Material Value Through Advanced Recycling Technologies

Once degradation has occurred during manufacturing and service, the effectiveness of circular polymer additive manufacturing depends on the ability of recycling technologies to recover not only material mass but also functional performance. Consequently, the objective of modern recycling strategies extends beyond simple waste diversion, aiming instead to preserve molecular integrity, restore engineering properties, and maximize the long-term value of polymer resources throughout multiple manufacturing lifecycles.

Mechanical recycling remains the most mature and widely adopted recovery pathway for thermoplastic polymers employed in additive manufacturing. Its industrial attractiveness arises from its relatively simple processing route, which typically involves collection, sorting, cleaning, shredding, remelting, and re-extrusion into new feedstocks. For extrusion-based additive manufacturing systems, this approach enables relatively efficient recovery of failed prints, support structures, production scrap, and end-of-life components with comparatively low environmental impact and infrastructure requirements. Nevertheless, repeated thermal and mechanical processing inevitably accelerates chain scission, oxidation, viscosity reduction, and molecular weight loss, progressively decreasing the mechanical performance and processing stability of recycled materials. As a result, mechanical recycling alone rarely enables indefinite closed-loop manufacturing without material stabilization or the incorporation of virgin polymer.

To overcome these limitations, increasing attention has been devoted to chemical recycling technologies capable of recovering the molecular building blocks of degraded polymers. Depolymerization, glycolysis, hydrolysis, methanolysis, pyrolysis, and solvent-assisted conversion processes enable polymers to be transformed into monomers or valuable chemical intermediates that can subsequently be repolymerized into materials exhibiting properties comparable to virgin feedstocks. Unlike mechanical recycling, these approaches partially or completely reset the molecular history accumulated during previous processing cycles. However, their broader industrial implementation remains constrained by higher energy consumption, greater process complexity, catalyst requirements, economic considerations, and the need for highly controlled operating conditions.

Solvent-based purification techniques have emerged as an intermediate strategy capable of combining material recovery with molecular preservation. Selective dissolution followed by purification and reprecipitation enables contaminants, pigments, fillers, and degradation products to be removed while maintaining the original polymer backbone largely intact. Such approaches are particularly attractive for high-value engineering polymers and complex additive manufacturing waste streams where conventional mechanical recycling would lead to unacceptable property deterioration. Nevertheless, solvent selection, recovery efficiency, environmental compatibility, and process economics remain critical challenges requiring further optimization before large-scale industrial adoption.

Emerging material regeneration technologies are further expanding the boundaries of circular manufacturing. Reactive extrusion, chain extension, molecular rebuilding, vitrimer chemistry, dynamic covalent polymer networks, and self-healing polymer systems seek not merely to recycle degraded materials but to actively restore their molecular architecture during reprocessing. These approaches represent a significant conceptual evolution from conventional recycling towards regenerative materials engineering, in which degradation accumulated during previous processing cycles can be partially reversed through controlled chemical or physicochemical reactions. Although many of these technologies remain at relatively early stages of development, they illustrate the growing transition from waste management towards functional material regeneration.

The effectiveness of any recycling technology is strongly influenced by the characteristics of the incoming material stream [144,145]. Polymer composition, molecular degradation state, contamination, moisture content, additive concentration, fibre reinforcement, pigment chemistry, and processing history collectively determine recovery efficiency and the quality of recycled feedstocks. Consequently, recycling performance should not be evaluated independently of previous manufacturing conditions but rather as the final outcome of the complete material lifecycle [145]. This observation reinforces the systems-oriented perspective developed throughout the present review.

From an industrial viewpoint, successful recycling technologies must simultaneously satisfy technical, environmental, and economic requirements. High material recovery efficiency alone does not guarantee circularity if excessive energy demand, complex logistics, poor scalability, or high operational costs compromise industrial feasibility. Likewise, recycling processes producing high-quality materials may remain commercially unattractive if collection systems, material traceability, or regulatory frameworks are insufficiently developed. Therefore, the selection of recycling technologies should be based on integrated lifecycle performance rather than isolated recovery efficiencies.

Future circular manufacturing systems will likely rely on combinations of complementary recycling technologies rather than individual recovery routes. Mechanical recycling may remain the preferred solution for relatively homogeneous thermoplastic waste streams, whereas chemical recycling and solvent-based regeneration may become increasingly important for complex, contaminated, or highly degraded materials [146,147,148]. Emerging regenerative polymer technologies may ultimately enable multiple high-value recovery cycles with significantly reduced losses in material functionality [149,150]. Consequently, hybrid recycling architectures capable of dynamically selecting the most appropriate recovery pathway according to material condition are expected to become a key component of next-generation circular additive manufacturing systems [151].

Overall, the reviewed evidence demonstrates that effective circularity depends not only on recovering polymer mass but on preserving, or restoring, the functional value embedded within polymer materials [152,153]. Recycling technologies should therefore be interpreted as integral elements of a broader lifecycle engineering strategy, where material design, manufacturing optimization, degradation mitigation, and intelligent recovery operate synergistically to maximize resource efficiency [154,155,156]. This integrated perspective naturally extends to the digital technologies discussed in the following section, where data-driven monitoring, predictive modelling, and intelligent traceability are examined as key enablers of future circular manufacturing systems.

### 4.4. Digital Intelligence and Traceability for Circular Polymer Additive Manufacturing

The successful implementation of circular polymer additive manufacturing increasingly depends on the integration of digital technologies capable of monitoring, predicting, and optimizing material behaviour throughout the complete product lifecycle. While advances in polymer engineering and recycling technologies have considerably improved material recovery, achieving truly closed-loop manufacturing requires continuous knowledge of material history, degradation state, processing conditions, and recovery potential. Consequently, digital intelligence has emerged as a fundamental enabler of next-generation circular manufacturing systems.

To operationalize this decision process, ICPAM is structured as a sequential multi-criteria decision-support methodology. First, the material, manufacturing history, degradation state, lifecycle performance, recovery potential, and industrial/digital readiness of the system are characterized using the ICPAM dimensions and the maturity assessment methodology described in Section 4.5 [157,158]. Second, technically infeasible alternatives are eliminated through constraint-based screening considering material compatibility, contamination, minimum property requirements, available recovery infrastructure, regulatory restrictions, and application-specific requirements. The remaining alternatives, such as direct reuse, repair, remanufacturing, mechanical recycling, chemical recycling, solvent-based recovery, downcycling, or final disposal, are then evaluated against a common set of technical, environmental, economic, circularity, and industrial criteria [159,160,161].

Because these criteria have different units and scales, their values should subsequently be normalized before aggregation. Criterion weights may initially be assigned equally when no defensible preference structure exists or, where application-specific priorities are available, derived through expert elicitation or established multi-criteria decision-making methods such as the Analytic Hierarchy Process (AHP). The feasible alternatives can then be ranked using an appropriate compensatory MCDM technique, such as TOPSIS, VIKOR, or another validated ranking method selected according to the decision context and data structure. Importantly, ICPAM does not prescribe a single universal MCDM algorithm; rather, it provides the lifecycle criteria, screening logic, and decision architecture within which an appropriate ranking methodology can be implemented.

A sensitivity analysis should subsequently examine whether the preferred alternative remains stable under reasonable changes in criterion weights and uncertain input data. If the ranking changes substantially, the decision should be regarded as sensitive and additional evidence should be collected before implementation. The selected strategy is finally monitored through the circularity and sustainability indicators defined within ICPAM, and the resulting performance data are returned to upstream material, design, manufacturing, service, and recovery decisions, thereby closing the lifecycle decision loop [162,163].

While the maturity methodology introduced in Section 4.5 evaluates the current state of an ICPAM system, the decision-support methodology described in this section uses that information, together with application-specific constraints and lifecycle performance criteria, to select among alternative circular strategies.

One of the principal limitations of current recycling practices lies in the absence of comprehensive material traceability. Once polymers enter manufacturing and subsequent recovery streams, information regarding their composition, processing history, thermal exposure, mechanical loading, recycling cycles, and degradation state is frequently lost. This lack of lifecycle information significantly complicates material classification, quality assessment, and recycling pathway selection, often resulting in conservative processing decisions or premature material disposal. Digital traceability addresses this limitation by preserving material identity throughout successive manufacturing and recovery operations.

Digital Twins have become one of the most promising technologies supporting lifecycle-oriented polymer management. By establishing virtual representations of physical materials and manufacturing processes, Digital Twins continuously integrate information from sensors, process monitoring systems, material databases, and operational feedback. These virtual models enable the prediction of thermal histories, degradation evolution, mechanical property retention, and remaining material lifespan before irreversible deterioration occurs. Consequently, Digital Twins support predictive decision-making, allowing manufacturing parameters and recycling strategies to be dynamically optimized according to the actual condition of each material batch.

Artificial intelligence (AI) and machine learning (ML) further expand these capabilities by extracting complex relationships from large manufacturing datasets. Through the analysis of process parameters, sensor signals, material characterization data, and recycling outcomes, AI algorithms can identify degradation patterns, predict property evolution, recommend optimal processing conditions, and classify recycled feedstocks according to their expected performance. Such predictive models reduce experimental trial-and-error while improving both manufacturing consistency and recycling efficiency.

Advanced sensing technologies constitute another essential component of intelligent circular manufacturing. Infrared thermography, optical imaging, spectroscopy, acoustic emission monitoring, force sensing, and embedded environmental sensors enable continuous observation of manufacturing conditions and material behaviour during production. When combined with real-time analytics, these sensing systems provide immediate feedback regarding process stability, defect formation, thermal anomalies, moisture content, or degradation onset, allowing corrective actions before significant material deterioration occurs.

Material traceability also benefits from recent developments in distributed digital infrastructures. Blockchain technologies, digital material passports, cloud manufacturing platforms, and standardized product identification systems facilitate secure recording of material composition, processing history, maintenance records, recycling cycles, and environmental performance. Such digital records improve transparency across manufacturing supply chains while enabling more reliable sorting, certification, regulatory compliance, and quality assurance for recycled materials entering subsequent production cycles.

The integration of these complementary digital technologies establishes the foundation for intelligent lifecycle management. Rather than reacting to material degradation after it has occurred, manufacturers can progressively transition towards predictive circularity, where degradation is continuously monitored, recycling decisions are optimized using real-time information, and material recovery pathways are selected according to the actual condition of each feedstock. This proactive approach considerably improves resource utilization while reducing unnecessary waste generation and virgin material consumption.

Despite their considerable potential, several challenges continue to limit the widespread industrial adoption of digital circular manufacturing [164,165]. Data interoperability between manufacturing platforms, standardization of material information, cybersecurity, data ownership, sensor reliability, computational requirements, and the integration of heterogeneous information sources remain significant barriers to implementation. Furthermore, many current digital solutions have been developed independently for manufacturing optimization or quality control, with relatively limited integration into comprehensive circular economy frameworks.

Future research should therefore prioritize the convergence of materials science, additive manufacturing, digital engineering, and artificial intelligence within unified decision-support architectures. Intelligent Digital Twins capable of continuously updating degradation models, AI-assisted adaptive process control, automated material classification, and standardized digital material passports are expected to become central components of future circular manufacturing ecosystems. By enabling continuous monitoring, predictive optimization, and lifecycle-wide traceability, digital intelligence has the potential to transform recycling from a reactive waste management activity into a fully integrated component of intelligent and sustainable manufacturing.

While the decision-support methodology in Figure 9 addresses which circular strategy should be selected, the maturity assessment introduced in Section 4.5 evaluates how comprehensively the ICPAM principles are currently implemented within the system.

### 4.5. Qualitative and Semi-Quantitative Assessment of ICPAM

To move ICPAM beyond a purely conceptual framework, a structured assessment methodology is proposed for evaluating the maturity and circular performance of polymer AM systems. The methodology combines qualitative lifecycle assessment with a semi-quantitative scoring approach aligned with the ICPAM dimensions identified in Figure 1. The objective is not to replace standardized LCA, LCC, reliability assessment, or material characterization methods, but to integrate their outputs within a common decision-support structure capable of identifying strengths, bottlenecks, and improvement priorities across the complete polymer AM lifecycle.

At the qualitative level, each ICPAM dimension is evaluated according to the availability, maturity, and integration of the corresponding practices and enabling mechanisms. Material Behaviour considers knowledge of polymer chemistry, degradation susceptibility, and recovery compatibility; Manufacturing-Induced Effects considers process control, thermal history, defect generation, and monitoring; Degradation Mechanisms addresses the characterization and control of property evolution across processing and service histories; Lifecycle Performance and Value Retention evaluates durability, reliability, repairability, maintainability, reuse potential, and traceability; Recycling and Recovery Pathways assesses the technical feasibility and value-retention capacity of available recovery routes; Circularity and Sustainability Performance considers material conservation, environmental impacts, recovery efficiency, and lifecycle economic performance; and Industrial and Digital Integration evaluates traceability, interoperability, qualification, reverse logistics, and adaptive decision-support capabilities.

For semi-quantitative implementation, each dimension *Di* may be assigned a normalized maturity score from 0 to 5, where 0 represents the absence of relevant practices or evidence, 1 an initial or ad hoc implementation, 2 a partially defined approach, 3 a systematically implemented approach, 4 an integrated and quantitatively monitored approach, and 5 a fully optimized, closed-loop, and adaptively managed condition. A dimension score can be obtained from the arithmetic mean of its constituent indicators after normalization to the same scale.

The overall ICPAM maturity index can then be expressed as

ICPAM Index = Σ w_i_*D_i_*, with Σ w_i_ = 1

where *Di* is the normalized score assigned to ICPAM dimension *i*, and *w_i_* represents its weighting factor. In the absence of application-specific evidence supporting differential weighting, equal weights should be adopted to minimize subjective bias. Alternatively, weighting factors may subsequently be established using expert elicitation, multicriteria decision-making methods, or application-specific priorities. Importantly, the overall index should always be interpreted together with the individual dimension scores, since a high aggregated value may otherwise conceal a critical weakness in one lifecycle stage.

The proposed assessment therefore enables both a qualitative maturity profile and a semi-quantitative comparison among materials, processes, products, or circular manufacturing scenarios. However, the scoring methodology should presently be regarded as a framework for subsequent empirical validation rather than as a fully validated predictive metric. Future studies should test its repeatability, sensitivity to weighting assumptions, correlation with independently measured LCA/LCC and material-performance indicators, and applicability across different polymer AM technologies and industrial sectors.

**Figure 11:**
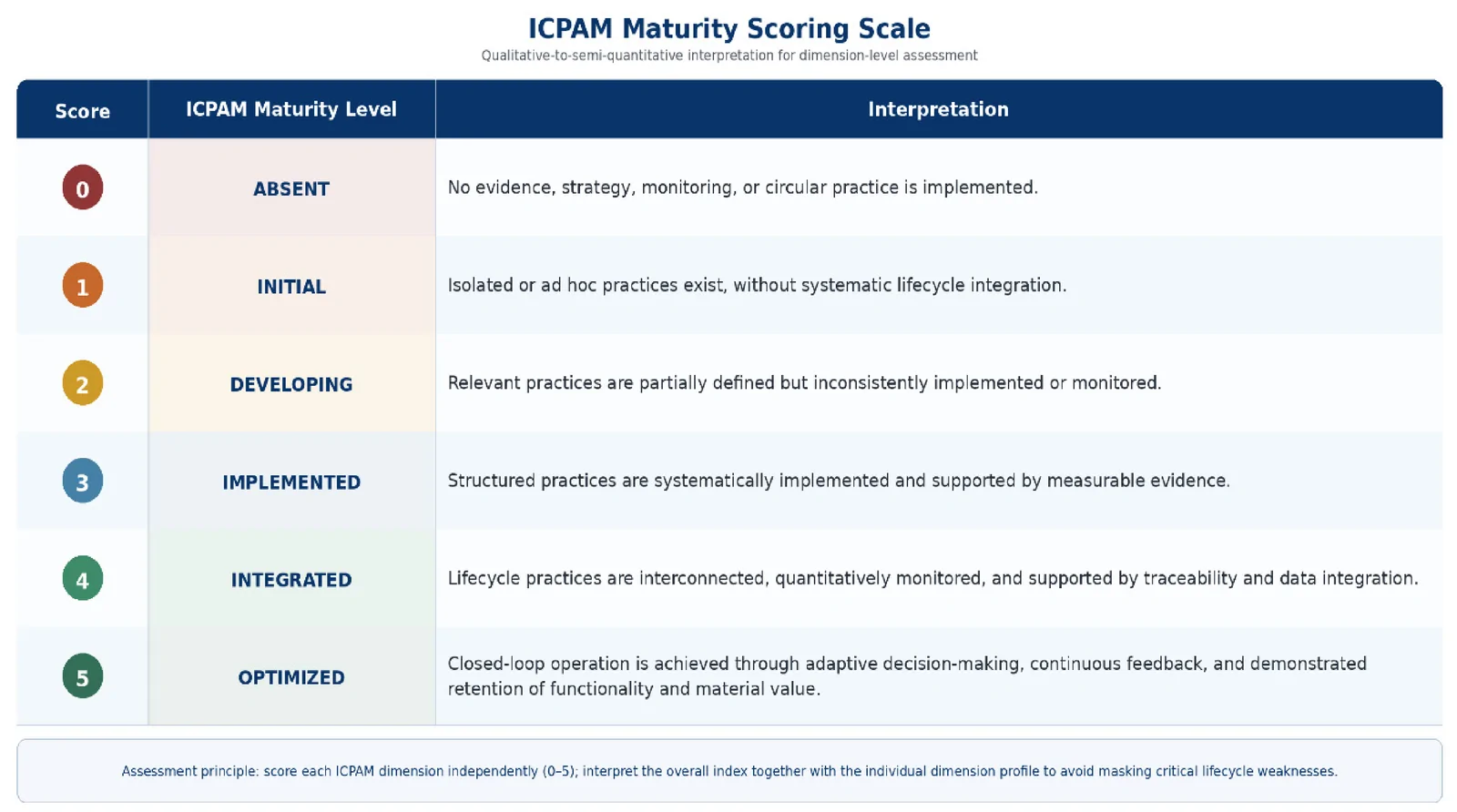
Qualitative and semi-quantitative maturity scoring scale proposed for the assessment of individual ICPAM dimensions.

To operationalize the proposed assessment methodology, a common maturity scale is required to ensure consistent interpretation across the ICPAM dimensions. Figure 11 therefore establishes a six-level scoring structure, ranging from the absence of systematic circular practices to fully optimized closed-loop implementation. The scale progressively captures the transition from isolated initiatives towards integrated, quantitatively monitored, and adaptively managed lifecycle practices.

The proposed scale enables each ICPAM dimension to be evaluated independently, thereby generating a multidimensional maturity profile rather than relying exclusively on a single aggregated indicator. This distinction is important because advanced performance in one dimension should not compensate for critical deficiencies elsewhere in the lifecycle. Accordingly, the overall ICPAM index should be interpreted together with the individual dimension scores, allowing technological bottlenecks, lifecycle discontinuities, and priority areas for improvement to be identified. At this stage, the 0–5 scale constitutes a structured assessment proposal derived from the ICPAM architecture; its discriminatory capacity, repeatability, and weighting strategy should be validated through future industrial case studies (Figure 11).

### 4.6. Enabling Industrial Implementation at Scale

Although substantial scientific progress has been achieved in polymer development, additive manufacturing technologies, recycling processes, and digital manufacturing, translating these advances into large-scale industrial practice remains a significant challenge. The implementation of circular polymer additive manufacturing extends far beyond technological feasibility, requiring the coordinated integration of manufacturing infrastructure, supply chain management, quality assurance, regulatory compliance, digital traceability, and economic sustainability [166]. Consequently, industrial circularity should be regarded as a systems engineering challenge rather than the direct consequence of isolated technological innovations [167,168].

One of the principal barriers to industrial implementation is the lack of standardized material qualification procedures for recycled feedstocks. While virgin polymers are generally supplied with well-defined specifications regarding mechanical performance, thermal properties, rheological behaviour, and processing windows, equivalent standards for recycled additive manufacturing materials remain comparatively limited. Variations in degradation history, contamination, additive concentration, moisture content, and molecular weight distribution introduce uncertainties that complicate certification and reduce confidence in industrial adoption. Establishing harmonized qualification protocols is therefore essential for enabling reliable use of recycled polymers in demanding engineering applications.

Supply chain organization represents another critical factor governing industrial scalability. Unlike conventional polymer manufacturing, additive manufacturing frequently generates decentralized waste streams distributed across multiple production facilities, service bureaus, research laboratories, and end users. Collecting, identifying, sorting, transporting, and reprocessing these heterogeneous material flows requires efficient reverse logistics networks capable of preserving feedstock quality while minimizing environmental and economic costs. Without robust collection and traceability systems, significant proportions of potentially recyclable materials continue to be diverted to low-value recovery routes or final disposal.

Economic competitiveness remains equally decisive for widespread industrial adoption. Although recycling generally reduces raw material consumption, the overall economic viability of circular manufacturing depends on the balance between collection costs, preprocessing requirements, material characterization, quality assurance, transportation, energy demand, equipment investment, and market acceptance of recycled products. In many cases, fluctuations in virgin polymer prices further complicate business models by reducing the financial attractiveness of recycled feedstocks. Consequently, industrial implementation requires economic frameworks capable of valuing not only material recovery but also resource resilience, supply security, regulatory compliance, and lifecycle sustainability [169,170].

Regulatory and certification requirements further influence the deployment of circular additive manufacturing, particularly in sectors such as aerospace, biomedical engineering, automotive, and consumer products, where stringent performance and traceability standards must be satisfied [171]. The absence of internationally harmonized regulations governing recycled additive manufacturing feedstocks often limits their acceptance in safety-critical applications despite demonstrated technical feasibility. Future regulatory frameworks will therefore need to incorporate lifecycle assessment methodologies, digital traceability systems, and standardized material qualification procedures to support broader industrial adoption.

Digital integration provides an important opportunity for overcoming many of these implementation barriers. Enterprise resource planning systems, Manufacturing Execution Systems (MES), Industrial Internet of Things (IIoT) platforms, Digital Twins, and digital material passports enable continuous monitoring of material flows across distributed manufacturing networks. By integrating production data, material characterization results, recycling histories, and quality records within unified digital environments, manufacturers can establish transparent and traceable circular supply chains capable of supporting industrial-scale resource management and regulatory compliance.

Industrial implementation also depends on workforce capabilities and organizational transformation. Engineers, designers, production managers, and sustainability specialists must increasingly operate within multidisciplinary environments where decisions concerning material selection, process optimization, digital monitoring, lifecycle assessment, and recycling are closely interconnected. Accordingly, education, professional training, and organizational change become strategic components of circular manufacturing, complementing technological innovation with the skills required to manage increasingly complex production ecosystems.

From a strategic perspective, the transition towards industrial-scale circularity requires replacing isolated optimization strategies with integrated lifecycle management [172]. Material design, manufacturing, degradation monitoring, recycling technologies, digital intelligence, logistics, regulatory compliance, and economic decision-making should operate as coordinated elements of a unified manufacturing ecosystem rather than as independent engineering activities. Within a comprehensive LCE perspective, this integration should also encompass distribution, service conditions, reliability, maintenance and repair strategies, component reuse or remanufacturing, and final end-of-life treatment, thereby ensuring that circularity decisions are optimized across the complete product lifecycle rather than exclusively at the manufacturing–recycling interface. Such integration enables continuous optimization of resource utilization while simultaneously improving productivity, environmental performance, product quality, and supply chain resilience [173].

Overall, the reviewed literature demonstrates that the future of circular polymer additive manufacturing will depend less on the invention of entirely new technologies than on the successful integration of existing innovations into coherent industrial ecosystems [174]. Achieving this objective requires coordinated advances in materials engineering, manufacturing science, digital technologies, standardization, logistics, and policy development. This systems-oriented perspective establishes the foundation for the emerging research directions discussed in the following section, where the principal scientific opportunities capable of accelerating the transition towards intelligent circular manufacturing are critically examined.

**Figure 12:**
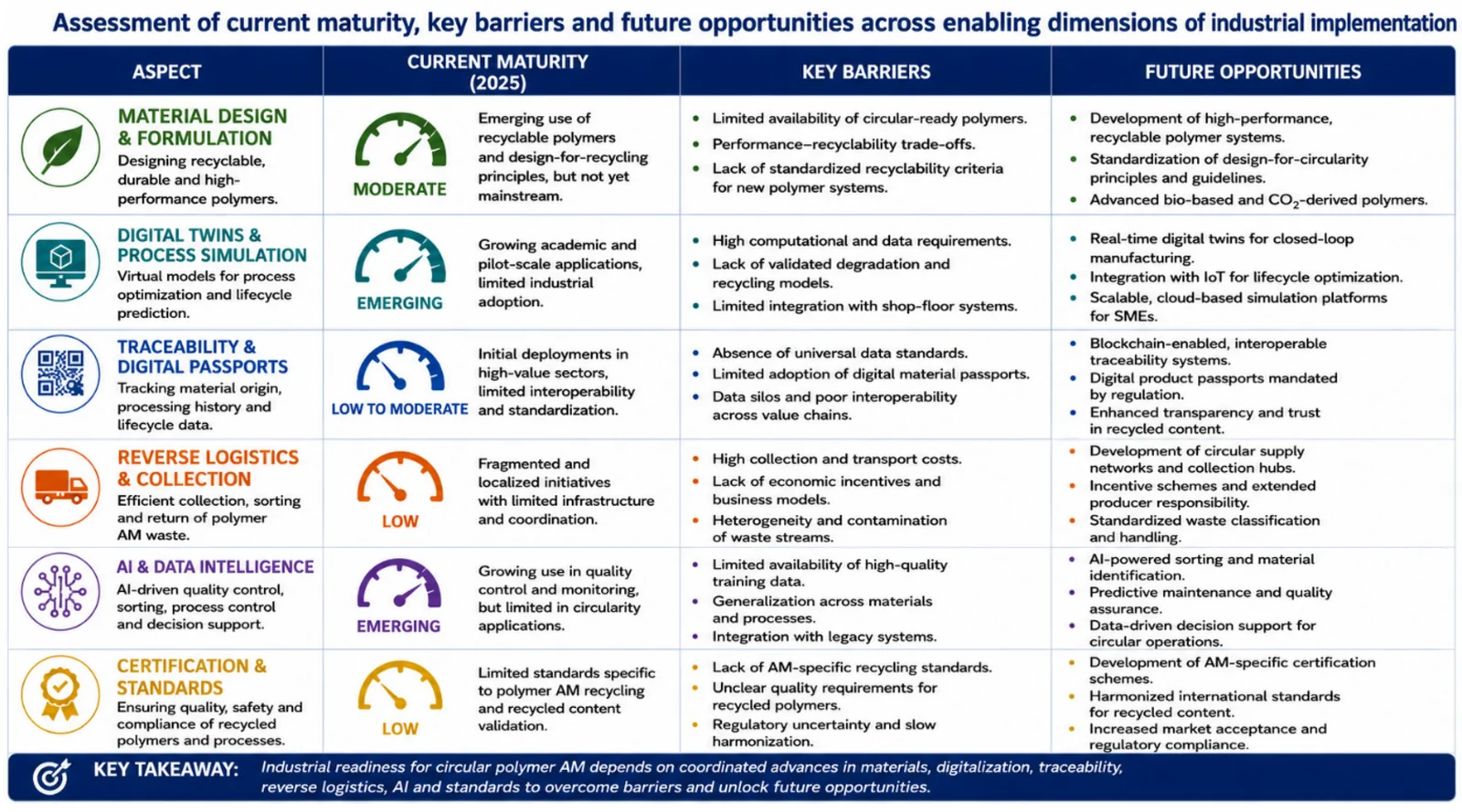
Industrial readiness matrix for circular polymer additive manufacturing.

Although considerable progress has been achieved in advancing circular polymer additive manufacturing technologies, their industrial implementation remains uneven across different enabling domains. To provide a structured assessment of the current landscape, Figure 12 summarizes the maturity level, principal implementation barriers, and future development opportunities associated with the key technological, digital, and organizational components required to establish industrial-scale circular manufacturing ecosystems.

As summarized in Figure 12, the successful industrial deployment of circular polymer additive manufacturing requires coordinated progress in materials development, digital technologies, traceability, reverse logistics, artificial intelligence, and certification frameworks. This integrated readiness assessment highlights the priority areas where scientific advances must be translated into industrial practice to enable large-scale implementation of intelligent circular manufacturing systems.

### 4.7. Future Scientific Directions

The rapid evolution of polymer additive manufacturing has substantially expanded the technological possibilities for implementing circular manufacturing systems. Nevertheless, the critical analysis presented throughout this review demonstrates that significant scientific challenges remain unresolved, particularly regarding the integration of materials science, manufacturing engineering, digital technologies, and lifecycle sustainability. Addressing these challenges requires a transition from incremental technological improvements towards multidisciplinary research capable of simultaneously advancing material performance, process intelligence, recycling efficiency, and industrial implementation.

A first priority concerns the development of polymer systems intrinsically designed for multiple manufacturing lifecycles. Current feedstocks remain largely optimized for primary processing rather than repeated recovery and reprocessing. Future research should therefore focus on molecular architectures capable of maintaining structural integrity under successive thermal and mechanical processing cycles while exhibiting enhanced resistance to oxidation, hydrolysis, and chain scission. Dynamic covalent polymers, vitrimer chemistries, reversible crosslinking systems, and adaptive polymer networks represent promising directions for extending material longevity within circular manufacturing environments [121,163,167]. However, most of these approaches remain at comparatively low technology-readiness levels for polymer additive manufacturing, with current evidence predominantly demonstrating material- or laboratory-scale feasibility rather than robust industrial deployment [163,167]. Their transition towards practical circular AM therefore requires further validation of printability, processing stability, property retention over multiple reprocessing cycles, scalability, reproducibility, lifecycle performance, and economic feasibility. Accordingly, these material platforms should presently be regarded as emerging research directions rather than mature alternatives to established polymer recovery technologies.

A second research frontier involves predictive degradation modelling across the complete additive manufacturing lifecycle. Although numerous studies have investigated isolated degradation mechanisms, comprehensive models capable of describing the cumulative evolution of material properties from feedstock production through manufacturing, service, recycling, and subsequent reuse remain relatively scarce [32,34,130,132]. Integrating experimental characterization with computational modelling, molecular simulation, and artificial intelligence offers significant opportunities for predicting material lifespan and optimizing recycling decisions before irreversible degradation occurs [105,108,109].

The convergence between additive manufacturing and digital engineering constitutes another major opportunity for future research. Intelligent Digital Twins capable of continuously updating material degradation models using real-time sensor data may fundamentally transform lifecycle management by enabling predictive control of manufacturing and recycling processes. Similarly, machine learning algorithms trained on large manufacturing datasets can support adaptive process optimization, automated material classification, defect prediction, and dynamic selection of recycling pathways according to the actual condition of recycled feedstocks.

Standardization represents an equally important scientific and industrial priority. Future investigations should contribute to the establishment of internationally harmonized methodologies for characterizing recycled feedstocks, evaluating degradation behaviour, assessing recyclability, and validating material performance throughout multiple manufacturing cycles. Such standardized protocols are essential for improving reproducibility, facilitating comparison between studies, supporting regulatory acceptance, and accelerating industrial deployment of circular polymer additive manufacturing [63,64,65,66,67,68,115].

Future research should also expand the application of lifecycle assessment methodologies beyond conventional environmental indicators. Integrated sustainability frameworks combining environmental performance, economic viability, technical functionality, resource criticality, social impact, and circularity metrics are required to provide a more comprehensive understanding of manufacturing sustainability [53,93,96,160,169,170,173]. Multi-criteria decision-support methodologies capable of balancing these complementary dimensions will become increasingly important as circular manufacturing systems continue to mature.

Another emerging research direction concerns the integration of circular manufacturing within distributed production ecosystems. The increasing decentralization of additive manufacturing raises important questions regarding reverse logistics, localized recycling infrastructures, digital material traceability, decentralized quality assurance, and resilient supply chain management [90,111,117,145]. Investigating these interactions will be essential for enabling regional and global circular manufacturing networks capable of efficiently recovering and redistributing polymer resources.

Perhaps the most significant long-term challenge lies in developing unified engineering frameworks capable of integrating material behaviour, manufacturing processes, degradation mechanisms, recycling technologies, digital intelligence, lifecycle assessment, and industrial decision-making within a single modelling environment [42,89,105,159,168,172]. Such integrated platforms would enable engineers to optimize circularity as a system-wide objective rather than as a sequence of independent technological improvements. Achieving this level of integration will require close collaboration between materials scientists, manufacturing engineers, chemists, computer scientists, environmental analysts, economists, and policymakers.

Overall, the future of circular polymer additive manufacturing will depend less on isolated technological breakthroughs than on the convergence of multiple scientific disciplines within intelligent lifecycle-oriented engineering systems [119,166,168,172]. The research directions identified throughout this review therefore suggest that the next generation of additive manufacturing will be characterized not only by improved materials and manufacturing technologies but also by predictive digital intelligence, regenerative material design, standardized circularity assessment, and integrated decision-support architectures capable of maximizing resource efficiency across the complete manufacturing lifecycle.

### 4.8. Towards an Intelligent Circular Polymer Additive Manufacturing Ecosystem

The evidence synthesized throughout this review demonstrates that achieving truly circular polymer additive manufacturing requires a fundamental transition from isolated technological improvements towards integrated lifecycle engineering. Although significant advances have been achieved independently in polymer development, additive manufacturing technologies, recycling processes, digital manufacturing, and sustainability assessment, these components continue to evolve largely as separate research domains [15,42,89,159,168]. Consequently, the principal challenge facing future manufacturing systems is no longer the development of individual technologies but their effective integration within intelligent circular production ecosystems.

The analytical framework proposed in this review indicates that circularity should be interpreted as a dynamic systems property emerging from continuous interactions between material design, manufacturing conditions, degradation behaviour, recycling technologies, digital intelligence, industrial infrastructure, and lifecycle management. Each of these elements influences the others through multiple feedback mechanisms operating throughout successive manufacturing and recovery cycles. As a result, improvements introduced at one stage of the lifecycle propagate across the entire manufacturing system, influencing both technical performance and long-term sustainability.

Within this integrated perspective, material design establishes the initial circularity potential by determining molecular stability, degradation resistance, processing behaviour, and recovery capability. Manufacturing processes subsequently preserve or compromise this potential through their influence on thermal history, mechanical loading, and process-induced material evolution [34,43,45,119,151]. Recycling technologies then attempt to recover the remaining functional value embedded within degraded materials, while digital intelligence continuously monitors material condition, predicts degradation trajectories, and optimizes recovery decisions based on real-time lifecycle information. Industrial implementation finally integrates these complementary technologies within economically viable, environmentally sustainable, and operationally resilient production systems.

This systems-oriented interpretation significantly expands the conventional understanding of circular manufacturing. Rather than viewing recycling as the final stage of product life, circularity becomes a continuously managed engineering objective extending from polymer synthesis to repeated material reintegration [106,111,112,113,159,172]. Consequently, future manufacturing systems should progressively evolve from linear production chains towards adaptive circular ecosystems, where material quality, process stability, resource efficiency, and environmental performance are continuously optimized through closed-loop feedback between physical manufacturing operations and digital decision-support architectures.

The convergence of additive manufacturing with artificial intelligence, Digital Twins, advanced sensing technologies, cloud manufacturing, blockchain-enabled traceability, and lifecycle assessment methodologies provide the technological foundation for this transformation [105,108,109]. Collectively, these technologies enable the creation of intelligent manufacturing environments capable of continuously identifying material degradation, dynamically adapting processing parameters, selecting optimal recycling pathways, and maximizing resource utilization throughout multiple production cycles. Such capabilities fundamentally redefine recycling as an active component of intelligent manufacturing rather than a downstream waste management operation.

The proposed roadmap therefore advocates a progressive transition towards Intelligent Circular Polymer Additive Manufacturing (ICPAM), an integrated manufacturing paradigm in which circularity is embedded throughout the entire lifecycle rather than addressed only after material degradation has occurred. Within this paradigm, polymer materials become continuously traceable engineering resources whose properties are monitored, predicted, preserved, recovered, and optimized through coordinated interaction between advanced materials engineering, digital technologies, manufacturing science, and circular economy principles.

From a scientific perspective, this integrated roadmap also provides a unified framework for future research. Instead of investigating materials, processes, degradation, recycling, digital technologies, or sustainability independently, future studies should increasingly examine the causal relationships existing between these domains. Such multidisciplinary integration will facilitate the development of predictive engineering methodologies capable of optimizing circularity simultaneously across molecular, process, product, factory, and supply chain levels.

Ultimately, the future of polymer additive manufacturing will depend not only on producing components with greater geometric complexity or improved mechanical performance, but on designing manufacturing systems capable of preserving material value throughout repeated lifecycles [107,112,113,131,170,174]. Achieving this objective requires replacing fragmented optimization strategies with intelligent, data-driven, and lifecycle-oriented manufacturing ecosystems in which circularity becomes an intrinsic engineering characteristic rather than an end-of-life objective. The integrated roadmap proposed in this review provides a conceptual foundation for supporting this transition while identifying the principal scientific and technological directions required to enable the next generation of sustainable polymer additive manufacturing.

**Figure 13:**
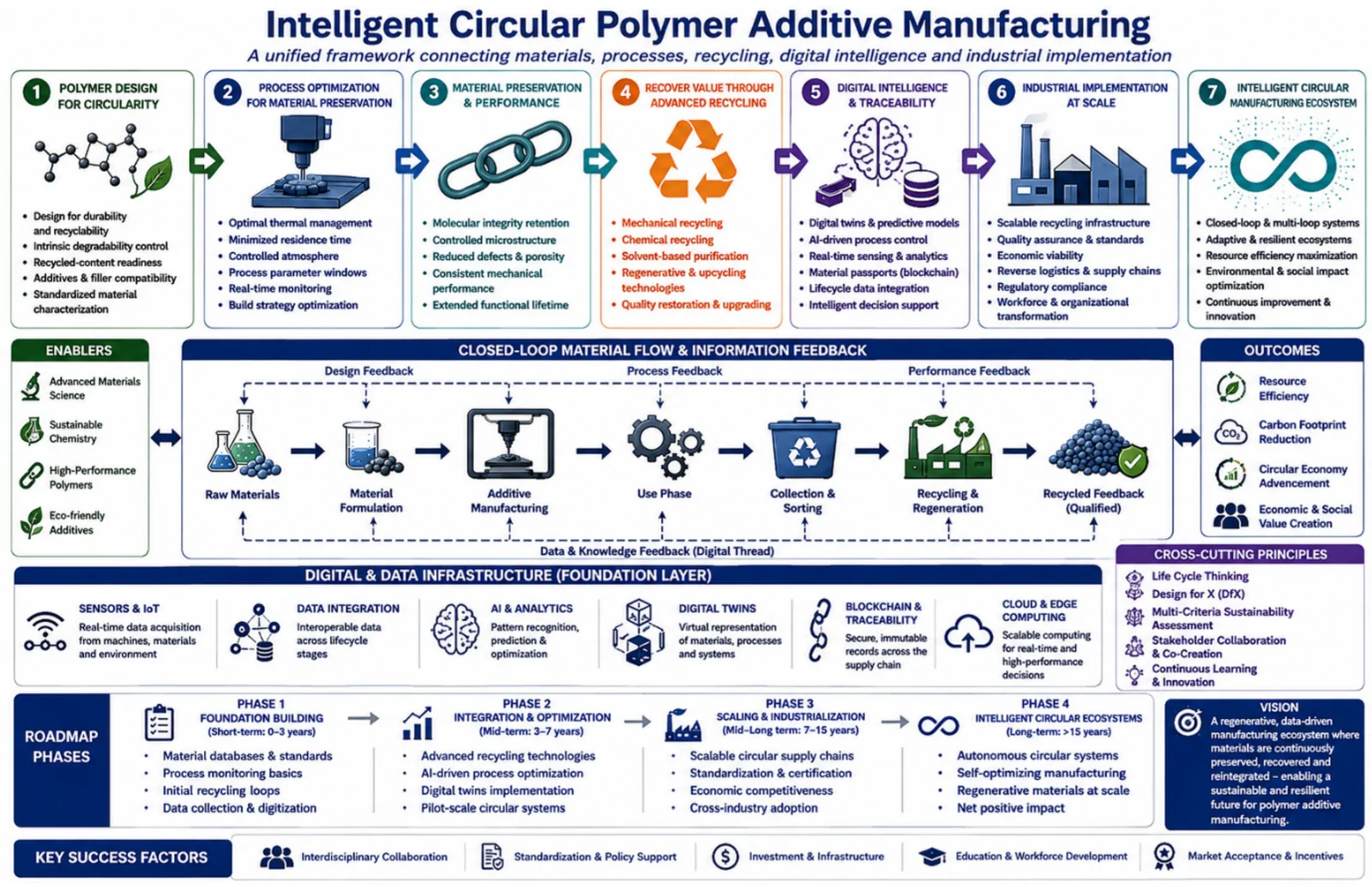
Integrated roadmap towards intelligent circular polymer additive manufacturing.

The critical analysis conducted throughout this review converges towards an integrated roadmap for enabling intelligent circular polymer additive manufacturing. As illustrated in Figure 13, the transition from conventional linear production to regenerative manufacturing ecosystems requires the coordinated interaction of material design, process optimization, advanced recycling technologies, digital intelligence, and industrial implementation within a unified lifecycle-oriented framework.

The proposed roadmap synthesizes the principal scientific insights identified throughout the literature into a unified systems-level framework, demonstrating that circularity is achieved through the continuous integration of materials engineering, manufacturing processes, digital technologies, and lifecycle management [42,119,159,166,168,172]. This perspective provides a strategic foundation for guiding both future research and the industrial implementation of sustainable polymer additive manufacturing.

## 5. Critical Synthesis and Future Perspectives

### 5.1. Principal Scientific Findings

The critical synthesis of the reviewed literature demonstrates that circular polymer additive manufacturing is governed by a significantly higher level of complexity than traditionally assumed. Rather than depending exclusively on recycling technologies or waste management practices, circularity emerges as the cumulative outcome of interconnected phenomena occurring throughout the complete material lifecycle [42,89,119,159,166,168]. Polymer chemistry, additive manufacturing processes, degradation mechanisms, recycling pathways, digital intelligence, and industrial infrastructure collectively determine the capacity of materials to retain functional value across successive manufacturing cycles.

One of the most consistent findings identified throughout the literature is that material degradation should be regarded as a lifecycle phenomenon rather than an isolated consequence of recycling. Molecular deterioration begins during feedstock production, progresses throughout additive manufacturing and service, and continues during repeated recovery operations. Consequently, the quality of recycled materials is determined by the complete processing history experienced by the polymer rather than by the efficiency of the recycling technology alone [32,34,40,43,121,130,132,142]. This observation fundamentally changes the interpretation of circularity, shifting attention from end-of-life recovery towards lifecycle-wide material preservation.

A second major finding concerns the decisive influence of manufacturing conditions on long-term recyclability. Additive manufacturing does not simply shape polymer components but actively modifies their molecular and microstructural characteristics through repeated thermal exposure, localized energy input, interlayer bonding, residual stress development, and environmental interactions. These process-induced transformations progressively alter degradation susceptibility and therefore predetermine the effectiveness of subsequent recycling operations [17,18,20,32,33,34,43,134,135,136]. Manufacturing optimization should therefore be interpreted not only as a productivity strategy but also as a critical mechanism for preserving future material value.

The reviewed studies further demonstrate that no universal recycling technology exists for polymer additive manufacturing. Mechanical recycling remains the most industrially mature solution for homogeneous thermoplastic waste streams, whereas chemical recycling, solvent-based purification, and emerging regenerative approaches provide greater opportunities for restoring material quality in more complex or highly degraded systems [46,47,52,78,97,98,99,100,101,146,147,148,149,150,151,152,153,154,155,156]. The selection of an appropriate recovery pathway is therefore strongly dependent on polymer chemistry, degradation history, contamination level, functional requirements, and economic constraints [48,49,50,51,144,151,175]. Consequently, recycling should be understood as an adaptive decision-making process rather than a standardized technological procedure.

Another important finding is the growing convergence between circular manufacturing and digital engineering. Technologies such as Digital Twins, artificial intelligence, machine learning, advanced sensing systems, blockchain-enabled traceability, and digital material passports increasingly provide the capability to monitor material evolution throughout the manufacturing lifecycle [105,108,109]. By enabling predictive degradation assessment, intelligent process optimization, and dynamic recycling pathway selection, these technologies transform circularity from a reactive waste management activity into a proactive lifecycle management strategy.

From an industrial perspective, the literature consistently indicates that technological innovation alone is insufficient to establish fully circular manufacturing systems. Successful implementation requires the simultaneous development of standardized material qualification protocols, robust quality assurance procedures, interoperable digital infrastructures, efficient reverse logistics, supportive regulatory frameworks, and economically viable business models [114,115,116,117,118,164,165,168]. Circularity therefore depends on the coordinated evolution of technical, organizational, environmental, and socioeconomic dimensions rather than on advances in individual manufacturing technologies.

Perhaps the most significant scientific finding emerging from this review is that polymer additive manufacturing should no longer be interpreted as a sequence of independent processing stages but rather as an integrated circular engineering system. Material design, manufacturing, degradation, recycling, digital intelligence, and industrial implementation continuously interact through multiple feedback mechanisms that collectively determine lifecycle performance [42,89,105,119,159,166,168,172]. This systems-level perspective provides a more comprehensive understanding of circular manufacturing while overcoming the fragmented approaches that continue to characterize much of the existing literature.

Collectively, these findings support a fundamental conceptual transition from technology-centred optimization towards lifecycle-oriented systems engineering. The integrated analytical framework proposed in this review demonstrates that maximizing circularity requires simultaneous optimization of material stability, manufacturing conditions, degradation control, recovery efficiency, digital traceability, and industrial integration. Circular polymer additive manufacturing should therefore be regarded not simply as a sustainable production strategy but as an intelligent engineering paradigm in which material value is continuously preserved, monitored, recovered, and regenerated throughout successive manufacturing lifecycles.

### 5.2. Convergence of Current Research Trends

The critical evaluation of the reviewed literature reveals a clear convergence in the scientific evolution of circular polymer additive manufacturing. Although individual studies continue to investigate diverse materials, processing technologies, recycling strategies, and sustainability assessment methods, the field is progressively moving towards integrated, lifecycle-oriented manufacturing paradigms [42,89,119,159,166,168,172]. This convergence reflects a broader transition from isolated technological optimization towards multidisciplinary engineering approaches capable of simultaneously addressing material performance, environmental sustainability, digitalization, and industrial implementation.

One of the most evident trends concerns the progressive shift from waste management towards material value preservation. Early research primarily focused on improving recycling efficiency and reducing polymer waste generation. More recent studies increasingly recognize that maintaining molecular integrity throughout successive manufacturing cycles provides greater long-term benefits than attempting to recover severely degraded materials at the end of life [34,43,107,112,113,131,151]. Consequently, increasing attention has been devoted to material design, degradation mitigation, and process optimization as complementary strategies for enhancing circularity.

A second area of convergence involves the integration of materials science with manufacturing process engineering. Rather than treating polymer chemistry and additive manufacturing as independent research domains, recent investigations increasingly examine the interactions between feedstock formulation, thermal history, microstructural evolution, and long-term recyclability [17,18,20,32,33,34,43,127,134,135,136]. This integrated perspective has significantly improved the understanding of how manufacturing conditions influence degradation behaviour and ultimately determine the quality of recycled feedstocks.

The literature also demonstrates growing consensus regarding the limitations of technology-specific solutions. Mechanical recycling, chemical recycling, solvent-based purification, and emerging regenerative technologies each provide distinct advantages under specific operating conditions, yet no single approach has emerged as universally applicable [46,47,78,97,98,99,100,101,146,147,148,149,150,151,152,153,154,155,156]. Instead, current research increasingly supports adaptive recycling strategies in which recovery pathways are selected according to polymer chemistry, degradation state, contamination level, functional requirements, and lifecycle objectives. This transition reflects a broader movement from standardized recycling procedures towards intelligent resource management.

Digitalization represents another major area of scientific convergence. Technologies such as Digital Twins, artificial intelligence, machine learning, advanced sensing, cloud manufacturing, blockchain, and digital material passports are progressively being incorporated into circular manufacturing research. Initially developed to improve productivity and quality control, these digital tools are increasingly recognized as key enablers of predictive lifecycle management, intelligent material traceability, and data-driven decision-making. Their integration with polymer recycling research illustrates the growing convergence between manufacturing science and digital engineering.

A further trend concerns the expansion of sustainability assessment beyond conventional environmental indicators. While early investigations primarily emphasized reductions in waste generation and material consumption, contemporary research increasingly incorporates energy efficiency, carbon emissions, economic feasibility, supply chain resilience, resource criticality, regulatory compliance, and social dimensions into comprehensive lifecycle evaluations. This multidimensional perspective reflects the recognition that circularity cannot be adequately assessed through isolated environmental metrics but requires balanced consideration of technical, economic, environmental, and societal performance.

Perhaps the most significant convergence identified throughout the literature is the emergence of systems thinking as the dominant scientific paradigm. Rather than analysing polymer materials, manufacturing processes, degradation mechanisms, recycling technologies, digital tools, or industrial implementation independently, an increasing number of studies advocate integrated frameworks capable of describing the interactions between these complementary domains. This evolution indicates a gradual transition from component-level optimization towards holistic lifecycle engineering, where circularity becomes an emergent property of the manufacturing ecosystem rather than the outcome of individual technological improvements.

Taken together, these converging research directions demonstrate that the future of circular polymer additive manufacturing will be shaped by the coordinated integration of advanced materials, intelligent manufacturing processes, adaptive recycling strategies, digital lifecycle management, and sustainable industrial ecosystems. The consistency with which these themes appear across the reviewed literature suggests that the field is progressively moving towards a unified scientific framework in which circularity is engineered through multidisciplinary collaboration rather than achieved through isolated technological advances.

### 5.3. Remaining Scientific Gaps

Despite the remarkable progress achieved in polymer additive manufacturing and circular economy research, the critical evaluation of the available literature reveals several fundamental scientific gaps that continue to limit the development of truly circular manufacturing systems. These gaps extend beyond individual technological limitations and reflect the fragmented manner in which materials science, manufacturing engineering, recycling technologies, digital systems, and sustainability assessment have traditionally evolved.

One of the most significant gaps concerns the absence of integrated lifecycle models capable of describing the cumulative evolution of polymer materials across successive manufacturing, service, recycling, and remanufacturing cycles. Most existing studies investigate isolated stages of the lifecycle, such as material characterization, process optimization, degradation behaviour, or recycling efficiency [15,42,89,119,159,166,168]. Consequently, the interactions linking these individual stages remain insufficiently understood, limiting the ability to predict long-term material performance under realistic industrial conditions.

Another important limitation involves the incomplete understanding of degradation accumulation mechanisms. Although thermal, oxidative, hydrolytic, photochemical, and mechanical degradation have been extensively investigated individually, considerably less attention has been devoted to their coupled evolution throughout repeated manufacturing and recycling cycles [32,34,36,40,43,121,130,132,141,142]. Predictive models capable of simultaneously considering multiple degradation pathways remain scarce, restricting the accurate estimation of material lifespan and recyclability after prolonged service and repeated processing.

The literature also reveals a lack of standardized methodologies for evaluating circularity in polymer additive manufacturing. Differences in experimental procedures, material characterization techniques, recycling protocols, degradation indicators, and lifecycle assessment methodologies make direct comparison between published studies particularly difficult [63,64,65,66,67,68,115,119,166]. This methodological heterogeneity limits reproducibility, complicates meta-analysis, and delays the establishment of internationally accepted benchmarks for assessing circular manufacturing performance.

A further scientific gap concerns the limited availability of predictive design methodologies for circular polymer systems. Most current material development strategies continue to prioritize printability and mechanical performance, while recyclability is frequently evaluated only after material formulation has been completed [17,18,26,32,127]. Future research should therefore shift towards predictive material engineering approaches in which molecular architecture, degradation resistance, processing stability, and recovery potential are simultaneously optimized during the earliest stages of material design.

Digital technologies also remain insufficiently integrated within current circular manufacturing research. Although Digital Twins, artificial intelligence, blockchain, smart sensing, and digital material passports have demonstrated considerable potential individually, relatively few studies investigate their coordinated application to lifecycle-wide material management [105,108,109]. Unified digital platforms capable of continuously tracking material condition, predicting degradation trajectories, optimizing processing parameters, and dynamically selecting recycling pathways remain largely conceptual, highlighting a significant opportunity for future multidisciplinary research.

Industrial implementation represents another area where important knowledge gaps persist. While laboratory-scale demonstrations of circular polymer additive manufacturing are becoming increasingly common, comparatively limited evidence is available regarding large-scale deployment, long-term process stability, supply chain integration, reverse logistics, regulatory compliance, and economic performance under industrial operating conditions [113,164,165,166,168,174]. Consequently, significant uncertainty remains concerning the practical scalability of many proposed circular manufacturing strategies.

The reviewed literature further indicates that sustainability assessments continue to be dominated by environmental considerations, whereas the interactions between environmental performance, economic viability, technical functionality, social acceptance, resource resilience, and regulatory constraints remain comparatively underexplored [53,93,96,116,117,118,160,165,169,170,173]. Developing comprehensive multi-dimensional assessment frameworks capable of balancing these complementary perspectives represents an important direction for future research.

Finally, perhaps the most fundamental scientific gap identified throughout this review is the absence of a unified theoretical framework capable of integrating materials science, additive manufacturing, degradation physics, recycling engineering, digital intelligence, lifecycle assessment, and industrial systems engineering within a common conceptual model. Existing investigations continue to address these domains largely independently, despite increasing evidence that circularity emerges from their continuous interaction [15,42,89,105,119,159,166,168,172]. Developing such integrated frameworks would substantially improve the predictive capability of future research while supporting the design of intelligent manufacturing ecosystems optimized for long-term material preservation and resource efficiency.

Overall, the remaining scientific gaps identified throughout this review indicate that the future evolution of circular polymer additive manufacturing will depend less on incremental improvements within individual disciplines than on the integration of complementary areas of expertise into coherent lifecycle-oriented engineering methodologies. Addressing these challenges will not only improve the technical performance of recycled materials but also accelerate the transition from isolated recycling solutions towards intelligent, predictive, and industrially scalable circular manufacturing systems.

### 5.4. Industrial and Sustainability Implications

The scientific evidence synthesized throughout this review demonstrates that the transition towards circular polymer additive manufacturing extends far beyond improvements in recycling efficiency. Instead, it requires a fundamental transformation of manufacturing systems in which material preservation, intelligent process control, digital lifecycle management, and sustainable resource utilization become integral components of industrial production. Consequently, circularity should be considered a strategic manufacturing objective capable of simultaneously enhancing environmental performance, economic competitiveness, and technological resilience.

From an industrial perspective, one of the most important implications concerns the progressive shift from linear material consumption towards lifecycle value retention. Conventional production systems typically regard polymer waste as an unavoidable by-product requiring downstream treatment, whereas circular additive manufacturing promotes the continuous preservation of material functionality throughout successive manufacturing cycles. This transition has the potential to reduce virgin polymer consumption, improve resource efficiency, stabilize raw material supply, and decrease production costs associated with material losses and waste management [90,111,112,113,131,165,168,170].

The findings of this review also highlight the increasing importance of integrating material science with intelligent manufacturing technologies. Advanced process monitoring, in situ sensing, Digital Twins, artificial intelligence, and predictive analytics enable manufacturers to anticipate material degradation, optimize processing conditions, and identify the most appropriate recovery pathways before significant quality deterioration occurs [105,108,109]. Such predictive lifecycle management strategies not only improve product consistency but also extend the functional lifespan of polymer feedstocks, contributing simultaneously to economic performance and environmental sustainability [34,43,107,131,138,142].

Another important industrial implication concerns the evolution of manufacturing business models. The widespread adoption of distributed additive manufacturing, localized recycling facilities, digital inventories, and closed-loop supply chains enables organizations to reduce transportation requirements, shorten production lead times, and improve resilience against disruptions in global material supply networks [90,111,113,117,145]. These decentralized manufacturing ecosystems are particularly relevant for sectors requiring rapid product customization, spare part production, and geographically distributed manufacturing capabilities.

The reviewed literature further indicates that industrial implementation will increasingly depend on robust digital traceability infrastructures. Digital material passports, blockchain-enabled data management, cloud-based manufacturing platforms, and interoperable information systems provide the transparency required to document material history, certify recycled feedstocks, support regulatory compliance, and facilitate quality assurance across multiple manufacturing cycles [105,108,109]. The integration of physical material flows with digital information networks therefore represents a critical enabler for large-scale industrial circularity.

From a sustainability perspective, the implications extend well beyond reductions in material waste. Effective circular polymer additive manufacturing contributes to lower energy demand associated with virgin material production, reduced greenhouse gas emissions, improved resource productivity, and enhanced resilience against critical raw material shortages [53,89,91,93,96,116,117,118,160,169,170]. Furthermore, by extending material service life and promoting multiple recovery cycles, circular manufacturing supports broader international sustainability objectives associated with climate neutrality, responsible production, and resource conservation [89,91,112,113,118,119].

Nevertheless, the literature consistently indicates that sustainability cannot be evaluated solely through environmental indicators. Economic viability, process reliability, product quality, regulatory acceptance, workforce readiness, and consumer confidence all influence the long-term success of circular manufacturing strategies [53,93,96,116,117,118,160,165,169,170,173]. Consequently, future industrial implementation should adopt multidimensional assessment frameworks capable of simultaneously balancing environmental, technical, economic, and societal objectives throughout the complete manufacturing lifecycle.

Collectively, these industrial and sustainability implications reinforce the need to interpret circular polymer additive manufacturing as a strategic engineering paradigm rather than a collection of isolated recycling technologies. Future manufacturing systems will increasingly depend on the seamless integration of advanced materials, intelligent manufacturing, adaptive recycling, digital infrastructure, and lifecycle-oriented decision-making. Organizations capable of implementing these interconnected capabilities will be better positioned to achieve higher resource efficiency, greater operational flexibility, enhanced environmental performance, and long-term industrial competitiveness within emerging circular economy frameworks.

### 5.5. Future Outlook

The evolution of circular polymer additive manufacturing is expected to extend well beyond incremental improvements in recycling technologies. The scientific evidence reviewed throughout this study suggests that the next generation of manufacturing systems will increasingly rely on the convergence of advanced materials, intelligent process control, digital lifecycle management, and systems engineering to establish manufacturing ecosystems capable of continuously preserving, recovering, and regenerating material value [42,89,119,159,166,168,172]. This transformation represents a fundamental shift from product-centred manufacturing towards intelligent resource-centred production paradigms.

Future advances are likely to be driven by the development of polymer materials specifically engineered for multiple manufacturing lifecycles. Rather than optimizing materials exclusively for printability or mechanical performance, next-generation feedstocks are expected to incorporate molecular architectures that facilitate repeated processing while maintaining structural integrity and functional performance. Dynamic covalent polymers, vitrimers, self-healing materials, reversible crosslinked networks, and bio-derived polymer systems are particularly promising candidates for enabling long-term material circularity without compromising engineering performance [76,88,121,163,167].

Digital technologies are expected to become increasingly central to lifecycle-oriented manufacturing. Artificial intelligence, Digital Twins, machine learning, advanced sensing systems, cloud manufacturing, and digital material passports will progressively evolve from isolated optimization tools into integrated decision-support platforms capable of continuously monitoring material condition, predicting degradation trajectories, recommending adaptive processing parameters, and identifying optimal recovery strategies throughout successive manufacturing cycles. Such capabilities will substantially enhance process reliability while enabling predictive rather than reactive circularity management [105,108,109].

Future manufacturing systems are also expected to exhibit significantly greater levels of autonomy and interoperability. Intelligent production environments will increasingly integrate additive manufacturing equipment, recycling infrastructure, quality assurance systems, logistics networks, and digital information platforms into interconnected manufacturing ecosystems capable of dynamically adapting to material condition, production demand, and sustainability objectives. This evolution will reinforce the role of polymer additive manufacturing as a key enabling technology for distributed, resilient, and resource-efficient industrial production [90,111,117,145].

From a research perspective, multidisciplinary collaboration will become increasingly important. Addressing the remaining scientific challenges identified throughout this review will require closer integration between polymer chemistry, materials science, manufacturing engineering, data science, artificial intelligence, environmental assessment, industrial engineering, and systems modelling [42,119,159,166,168,172]. The future of circular manufacturing therefore depends not only on technological innovation but also on the development of common scientific languages, standardized methodologies, interoperable datasets, and collaborative research frameworks capable of bridging traditionally independent disciplines.

Future progress will likewise depend on the establishment of internationally harmonized standards supporting material qualification, recyclability assessment, digital traceability, lifecycle modelling, and sustainability evaluation [63,64,65,66,67,68,115,164,166]. Such standardization will facilitate industrial adoption, improve reproducibility across research laboratories, enhance regulatory confidence, and accelerate the commercialization of circular manufacturing technologies across multiple industrial sectors.

Ultimately, the long-term vision emerging from this review extends beyond the implementation of closed-loop recycling systems. Instead, the literature increasingly supports the development of intelligent circular manufacturing ecosystems in which material evolution is continuously monitored, predicted, optimized, and regenerated throughout the complete product lifecycle [105,109,119,159,168,172]. Within such ecosystems, circularity becomes an intrinsic engineering characteristic designed into materials, manufacturing processes, digital infrastructures, and industrial business models from the earliest stages of product development.

The critical synthesis developed throughout this review converges towards a unified scientific perspective in which circular polymer additive manufacturing is interpreted as an intelligent, lifecycle-oriented engineering ecosystem. To consolidate this conceptual evolution, Figure 8 presents the proposed Intelligent Circular Polymer Additive Manufacturing (ICPAM) framework, integrating material design, manufacturing optimization, adaptive recycling, digital intelligence, and lifecycle management into a single systems-level architecture capable of supporting future research and industrial implementation.

As illustrated in Figure 10, the proposed ICPAM framework redefines circular polymer additive manufacturing as an interconnected engineering ecosystem in which material value is continuously preserved, monitored, recovered, and regenerated throughout successive lifecycle stages. By integrating materials science, manufacturing engineering, recycling technologies, digital intelligence, and industrial systems within a unified conceptual model, this framework constitutes the principal scientific contribution of the present review and provides a strategic foundation for the next generation of intelligent circular manufacturing systems.

Looking ahead, the successful realization of this vision will require a transition from technology-driven innovation towards lifecycle-driven engineering. The integrated framework proposed throughout this review provides a conceptual foundation for this transition by demonstrating that sustainable polymer additive manufacturing depends on the coordinated interaction of material design, manufacturing optimization, degradation control, adaptive recycling, digital intelligence, and industrial integration. As these domains continue to converge, circular polymer additive manufacturing is expected to evolve from an emerging sustainability strategy into a mature manufacturing paradigm capable of supporting resilient, low-carbon, and resource-efficient production systems for future generations.

**Figure 14:**
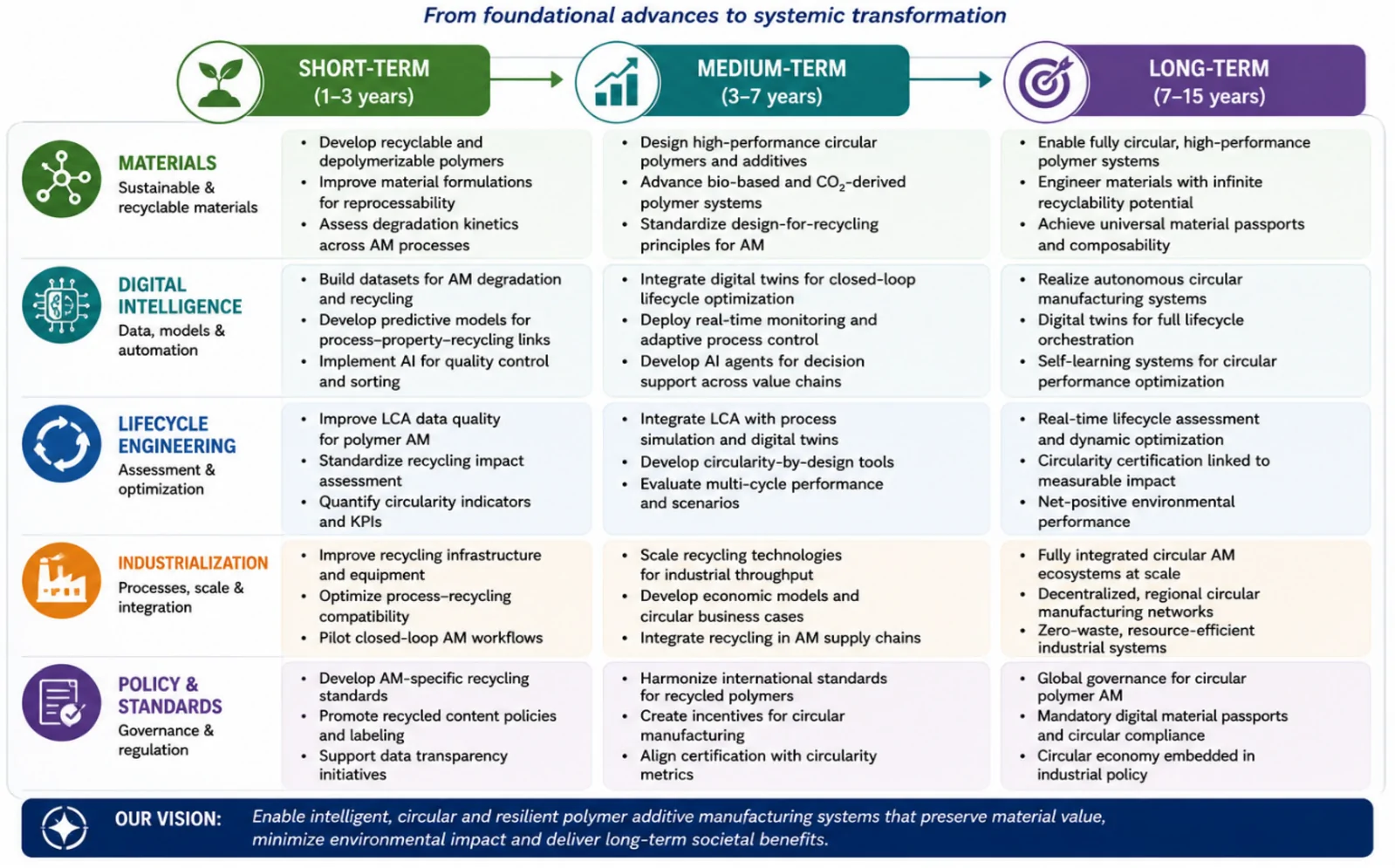
Research priorities map for intelligent circular polymer additive manufacturing.

The critical analysis performed throughout this review not only identifies the current state of knowledge but also reveals a clear trajectory for the future evolution of circular polymer additive manufacturing. To consolidate these perspectives, Figure 14 presents a structured research priorities map that organizes the principal scientific and technological challenges according to short-, medium-, and long-term development horizons, providing a strategic roadmap for future research and industrial innovation.

**Figure 15:**
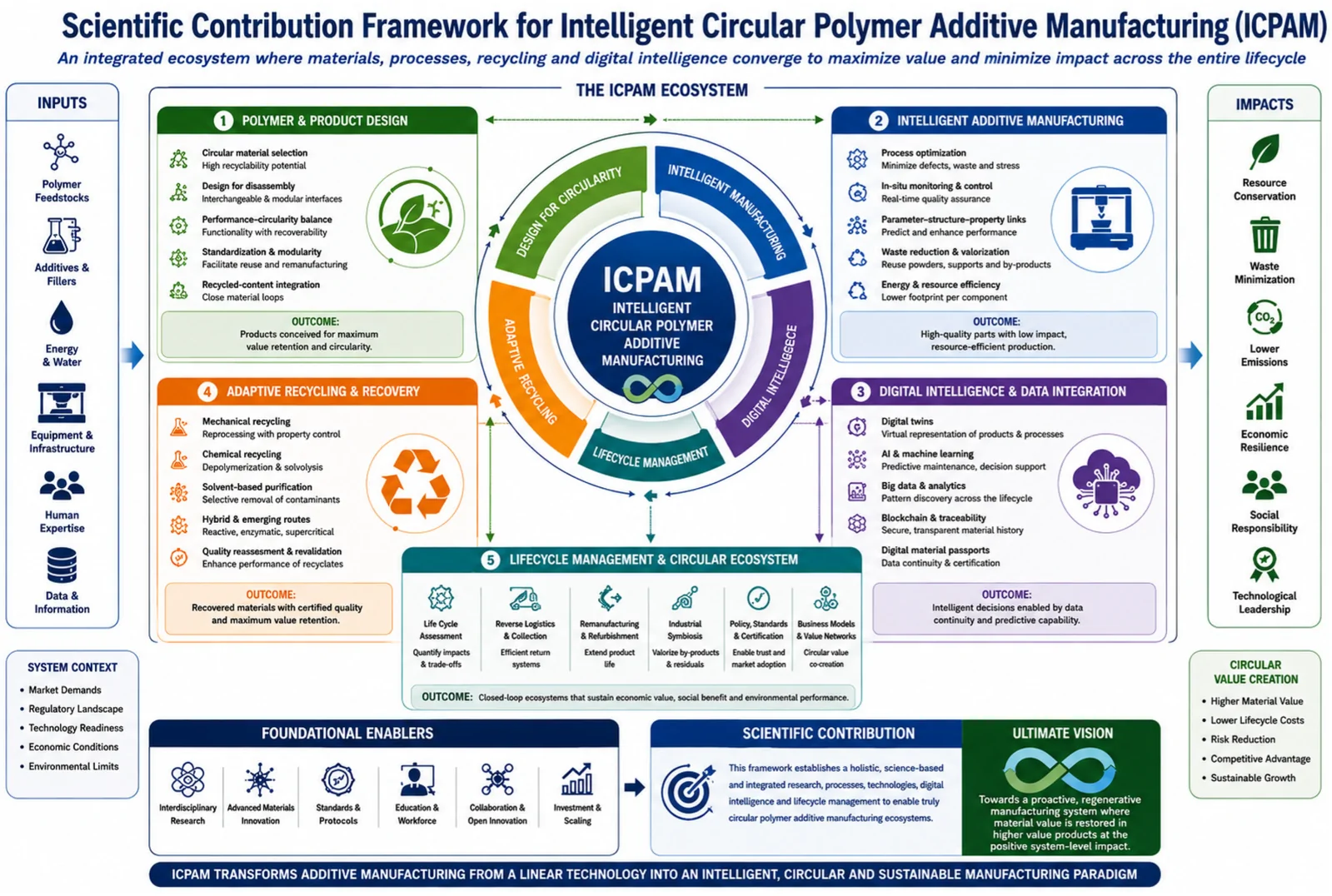
The Intelligent Circular Polymer Additive Manufacturing (ICPAM) framework.

As illustrated in Figure 15, the successful transition towards intelligent circular polymer additive manufacturing will require coordinated advances in materials engineering, digital intelligence, lifecycle assessment, industrial implementation, and regulatory frameworks. This temporal perspective emphasizes that achieving fully circular manufacturing ecosystems depends on the progressive integration of complementary scientific disciplines, providing a strategic foundation for the concluding remarks and future vision presented in the final chapter.

## 6. Conclusions

This critical review has demonstrated that circular polymer additive manufacturing cannot be adequately understood through the isolated analysis of recycling technologies or material recovery processes. Instead, circularity emerges as a systems-level engineering property resulting from the continuous interaction between polymer chemistry, additive manufacturing processes, degradation mechanisms, recycling pathways, digital intelligence, industrial implementation, and lifecycle management. This integrated perspective represents a fundamental shift from conventional end-of-life recycling approaches towards intelligent lifecycle engineering.

The comprehensive analysis of the reviewed literature revealed that material degradation begins long before recycling takes place and evolves cumulatively throughout successive manufacturing, service, recovery, and remanufacturing cycles. Consequently, preserving material functionality requires coordinated optimization of feedstock design, process parameters, degradation mitigation, adaptive recycling strategies, and digital monitoring technologies rather than relying solely on improvements in recycling efficiency. This finding redefines circularity as a proactive material preservation strategy instead of a reactive waste management solution.

The review further demonstrated that no universal recycling pathway is capable of addressing the diversity of polymer additive manufacturing systems. Mechanical recycling, chemical recycling, solvent-based purification, reactive processing, and emerging regenerative approaches each provide complementary capabilities depending on polymer chemistry, degradation history, application requirements, and economic constraints. Future circular manufacturing systems will therefore require adaptive decision-making frameworks capable of dynamically selecting the most appropriate recovery strategy according to lifecycle-specific conditions.

Another important conclusion concerns the transformative role of digital technologies in enabling intelligent circular manufacturing. Artificial intelligence, Digital Twins, machine learning, advanced sensing systems, blockchain-enabled traceability, and digital material passports collectively provide the technological foundation required for predictive lifecycle management. Their integration enables continuous monitoring of material evolution, optimization of manufacturing conditions, and intelligent recovery planning, thereby significantly extending material service life while improving manufacturing efficiency and sustainability.

Beyond technological advances, this review highlights that the successful industrial implementation of circular polymer additive manufacturing depends on the coordinated development of standardized qualification methodologies, interoperable digital infrastructures, robust quality assurance procedures, efficient reverse logistics, supportive regulatory frameworks, and economically sustainable business models. Circularity should therefore be recognized as a multidisciplinary engineering challenge requiring simultaneous advances in materials science, manufacturing engineering, digital technologies, industrial systems, and sustainability assessment.

Perhaps the principal scientific contribution of this review is the proposal of an integrated conceptual framework in which circularity is interpreted as an emergent property of intelligent manufacturing ecosystems rather than the outcome of individual technological improvements. By synthesizing evidence across polymer materials, additive manufacturing processes, degradation mechanisms, recycling technologies, digital intelligence, and industrial implementation, the proposed framework provides a unified perspective capable of supporting future research, technology development, and industrial decision-making.

Looking forward, the future of polymer additive manufacturing will increasingly depend on the transition from technology-centred innovation towards lifecycle-oriented systems engineering. Materials specifically designed for multiple recovery cycles, predictive degradation modelling, intelligent manufacturing platforms, adaptive recycling systems, and digitally connected industrial ecosystems are expected to become fundamental components of next-generation circular production systems. As these technologies continue to converge, polymer additive manufacturing has the potential to evolve from an emerging sustainable manufacturing strategy into a mature engineering paradigm supporting resilient, resource-efficient, and low-carbon industrial production.

In conclusion, this review demonstrates that achieving true circularity requires more than improving individual manufacturing technologies; it demands the coordinated integration of materials engineering, process optimization, digital intelligence, lifecycle management, and industrial systems thinking. The transition towards Intelligent Circular Polymer Additive Manufacturing (ICPAM) proposed throughout this work provides a coherent scientific foundation for this evolution and establishes a strategic roadmap capable of guiding both future research and the industrial implementation of circular manufacturing ecosystems.

## Data Availability

No new data were created or analyzed in this study. Data sharing is not applicable to this article.

## Supplementary Materials

The following supporting information can be downloaded at: https://www.mdpi.com/article/10.3390/polym18172161/s1, Table S1: Preferred Reporting Items for Systematic reviews and Meta-Analyses extension for Scoping Reviews (PRISMA-ScR) Checklist. Reference [176] is cited in Supplementary Materials.
