# Supplementary material for: Intelligent Circular Polymer Additive Manufacturing: From Recycling Pathways to Lifecycle Engineering
Source: Polymers (Basel). 2026 Sep 4;18(17):2161. doi: 10.3390/polym18172161 (PMC13567976; doi:10.3390/polym18172161)
Supplement: Supplementary file 1 [file polymers-18-02161-s001.zip › polymers-4525182-supplementary.pdf]

Table S1: Preferred Reporting Items for Systematic reviews and Meta-Analyses extension for Scoping Reviews (PRISMA-ScR) Checklist

SECTION	ITEM	PRISMA-ScR CHECKLIST ITEM	REPORTED ON PAGE #
**TITLE**			
Title	1	The manuscript is identified as a Review and is titled "Intelligent Circular Polymer Additive Manufacturing: From Recycling Pathways to Lifecycle Engineering".	p. 1
**ABSTRACT**			
Structured summary	2	The abstract summarises the background, review scope, PRISMA-based selection process, corpus of 175 studies, principal systems-level findings, and the proposed Intelligent Circular Polymer Additive Manufacturing (ICPAM) framework.	p. 2
**INTRODUCTION**			
Rationale	3	The introduction explains why manufacturing-stage material efficiency is insufficient to establish circularity and identifies the fragmented treatment of degradation, recovery, lifecycle assessment, and industrial implementation as the principal knowledge gap.	Section 1, pp. 2-5
Objectives	4	The review aims to integrate polymer behaviour, manufacturing-induced evolution, degradation, value retention, recovery pathways, sustainability assessment, and digital/industrial enablers. Five research questions define the detailed analytical objectives.	Section 1, pp. 4-5; Section 2.2, pp. 8-10
**METHODS**			
Protocol and registration	5	No separate review protocol or registration number is reported. The PRISMA-based review design and the six-dimensional ICPAM scientific framework are described in the Methods.	Not reported; review design: Section 2.1, pp. 5-8
Eligibility criteria	6	Eligibility criteria specify publication type, 2015-2025 date range, polymer-based AM processes, circularity-related scope, acceptable evidence types, and explicit exclusion criteria.	Section 2.3, p. 12
Information sources*	7	The information sources comprise Scopus, Web of Science Core Collection, ScienceDirect, MDPI, and supplementary reference-list screening. The publication coverage period is reported, but the date of the last search is not stated.	Section 2.2, pp. 10-11
Search	8	The search strategy reports three concept domains, their principal keywords, and use of Boolean AND/OR combinations to connect additive manufacturing, polymer, and circularity/recycling terminology.	Section 2.2, pp. 10-11
Selection of sources of evidence†	9	Records underwent title/abstract screening, full-text assessment against predefined eligibility criteria, critical appraisal, and removal of redundant evidence before qualitative synthesis.	Section 2.4, pp. 13-14

SECTION	ITEM	PRISMA-ScR CHECKLIST ITEM	REPORTED ON PAGE #
Data charting process‡	10	A standardised protocol was applied to all eligible publications to chart evidence across six analytical categories spanning manufacturing, materials, degradation, recovery, performance, and sustainability/lifecycle indicators.	Section 2.5, pp. 14-16
Data items	11	Data items include AM technology and conditions; polymer composition and properties; degradation mechanisms; recovery strategy and efficiency; property retention; and environmental, economic, scalability, logistics, and infrastructure indicators.	Table 2, pp. 9-10; Section 2.5, pp. 14-15
Critical appraisal of individual sources of evidence§	12	Individual sources were qualitatively appraised for methodological rigour, technical relevance, evidence strength, and scientific contribution. A numerical score was not used because of substantial evidence heterogeneity.	Section 2.5, pp. 15-16
Synthesis of results	13	Evidence was critically compared rather than statistically pooled, then integrated through a four-layer analytical framework linking material fundamentals, manufacturing effects, recycling-induced degradation, and circularity performance.	Sections 2.5-2.6, pp. 15-18
**RESULTS**			
Selection of sources of evidence	14	The PRISMA flow reports 3,214 identified records, 802 duplicates removed, 2,412 records screened, 286 full texts assessed, 111 full texts excluded, and 175 studies included in the qualitative synthesis.	Section 2.4, pp. 13-14; Figure 3, p. 14
Characteristics of sources of evidence	15	Evidence characteristics are synthesised thematically by polymer/feedstock family, AM process, degradation mechanism, recovery pathway, circularity constraint, enabling technology, and industrial implementation dimension.	Sections 3-4, pp. 18-47
Critical appraisal within sources of evidence	16	Source-level critical-appraisal results are not reported separately; the qualitative four-dimension appraisal approach used to interpret the evidence is described in the Methods.	Not separately reported; appraisal method: Section 2.5, pp. 15-16
Results of individual sources of evidence	17	Findings from individual sources are cited and critically compared throughout the thematic analysis of materials, process effects, degradation, recovery, digital enablers, industrial readiness, and sustainability implications.	Sections 3-5, pp. 18-55
Synthesis of results	18	The synthesis connects the scientific evidence to the ICPAM lifecycle architecture, maturity model, industrial-readiness matrix, decision-support logic, integrated roadmap, and research priorities.	Section 3.6, pp. 30-31; Sections 4.8-5, pp. 45-55
**DISCUSSION**			
Summary of evidence	19	The critical synthesis summarises the principal findings, convergence of research trends, remaining scientific gaps, industrial and sustainability implications, and future outlook in relation to the five research questions.	Section 5, pp. 47-55

SECTION	ITEM	PRISMA-ScR CHECKLIST ITEM	REPORTED ON PAGE #
Limitations	20	Heterogeneous system boundaries, functional units, datasets, and assessment methods are identified as preventing direct numerical aggregation or meta-analysis. Remaining evidence and implementation gaps are also discussed.	Section 2.5, p. 15; Section 5.3, pp. 50-52
Conclusions	21	The manuscript concludes that circularity is an emergent lifecycle outcome, presents ICPAM as an integrated systems-level framework, and identifies research and industrial priorities for adaptive, digitally enabled circular polymer AM.	Sections 5.5-6, pp. 53-57
**FUNDING**			
Funding	22	The manuscript reports support from the Drivolution - Transition to the factory of the future project (Project Code 02/C05-i01.02/2022, Project no. 23), supported by European Structural and Investment Funds within the Portugal 2020 programme.	Funding, p. 58

JBI = Joanna Briggs Institute; PRISMA-ScR = Preferred Reporting Items for Systematic reviews and Meta-Analyses extension for Scoping Reviews.

* Where *sources of evidence* (see second footnote) are compiled from, such as bibliographic databases, social media platforms, and Web sites.

† A more inclusive/heterogeneous term used to account for the different types of evidence or data sources (e.g., quantitative and/or qualitative research, expert opinion, and policy documents) that may be eligible in a scoping review as opposed to only studies. This is not to be confused with *information sources* (see first footnote).

‡ The frameworks by Arksey and O'Malley (6) and Levac and colleagues (7) and the JBI guidance (4, 5) refer to the process of data extraction in a scoping review as data charting.

§ The process of systematically examining research evidence to assess its validity, results, and relevance before using it to inform a decision. This term is used for items 12 and 19 instead of "risk of bias" (which is more applicable to systematic reviews of interventions) to include and acknowledge the various sources of evidence that may be used in a scoping review (e.g., quantitative and/or qualitative research, expert opinion, and policy document).

From: Tricco AC, Lillie E, Zarin W, O'Brien KK, Colquhoun H, Levac D, et al. PRISMA Extension for Scoping Reviews (PRISMAScR): Checklist and Explanation. Ann Intern Med. 2018;169:467–473. doi: 10.7326/M18-0850.
